# Ethylene signaling is essential for mycorrhiza-induced resistance against chewing herbivores in tomato

**DOI:** 10.1093/jxb/eraf053

**Published:** 2025-02-08

**Authors:** Javier Lidoy, Javier Rivero, Živa Ramšak, Marko Petek, Maja Križnik, Victor Flors, Juan A Lopez-Raez, Ainhoa Martinez-Medina, Kristina Gruden, Maria J Pozo

**Affiliations:** Department of Soil and Plant Microbiology, Estación Experimental del Zaidín, CSIC, Granada, Spain; Department of Plant Physiology and Biochemistry, University of Hohenheim, Stuttgart, Germany; Department of Soil and Plant Microbiology, Estación Experimental del Zaidín, CSIC, Granada, Spain; Department of Cell Biology, Genetics and Physiology, Instituto de Hortofruticultura Subtropical y Mediterránea ‘La Mayora’ (IHSM-UMA-CSIC), Málaga, Spain; Department of Biotechnology and Systems Biology, National Institute of Biology, Ljubljana, Slovenia; Department of Biotechnology and Systems Biology, National Institute of Biology, Ljubljana, Slovenia; Department of Biotechnology and Systems Biology, National Institute of Biology, Ljubljana, Slovenia; Plant Immunnity and Biochemistry Laboratory, Department of Biology, Biochemistry and Natural Sciences, Universitat Jaume I, Castelló de la Plana, Spain; Department of Soil and Plant Microbiology, Estación Experimental del Zaidín, CSIC, Granada, Spain; Department of Soil and Plant Microbiology, Estación Experimental del Zaidín, CSIC, Granada, Spain; Department of Biotechnology and Systems Biology, National Institute of Biology, Ljubljana, Slovenia; Department of Soil and Plant Microbiology, Estación Experimental del Zaidín, CSIC, Granada, Spain; Nanjing Agricultural University, China

**Keywords:** Chewing herbivores, defense priming, ethylene, hormonal crosstalk, jasmonate, mycorrhiza-induced resistance (MIR)

## Abstract

Arbuscular mycorrhizal (AM) symbiosis can prime plant defenses, leading to mycorrhiza-induced resistance (MIR) against different attackers, including insect herbivores. Still, our knowledge of the complex molecular regulation leading to MIR is very limited. Here, we showed that the AM fungus *Funneliformis mosseae* protects tomato plants against two different chewing herbivores, *Spodoptera exigua* and *Manduca sexta*. We explored the underlying molecular mechanism through genome-wide transcriptional profiling, bioinformatics network analyses, and functional bioassays. Herbivore-triggered jasmonate (JA)-regulated defenses were primed in leaves of mycorrhizal plants. Likewise, ethylene (ET) biosynthesis and signaling were also higher in leaves of mycorrhizal plants both before and after herbivory. We hypothesized that fine-tuned ET signaling is required for the primed defense response leading to MIR. ET is a complex regulator of plant responses to stress and is generally considered a negative regulator of plant defenses against herbivory. However, ET-deficient or insensitive lines did not show AM-primed JA biosynthesis or defense response, and were unable to develop MIR against any of the herbivores. Thus, we demonstrate that hormone crosstalk is central to the priming of plant immunity by beneficial microbes, with ET fine-tuning being essential for the primed JA biosynthesis and boosted defenses leading to MIR in tomato.

## Teaser

Hormonal crosstalk is essential for mycorrhiza-induced resistance against chewing herbivores. Specifically, ethylene signaling boosts jasmonate biosynthesis and defensive response in mycorrhizal plants.

## Introduction

Upon herbivory, plants activate an extensive arsenal of defensive mechanisms including toxic, antidigestive, and repellent compounds (Broadway and Duffey, 1988; Duffey and Stout, 1996; Erb and Reymond, 2019). The jasmonate (JA) signaling pathway plays a central role in the transcriptional reorganization and the induction of defenses in the process (Wasternack and Hause, 2013; Erb and Reymond, 2019; Wang *et al.*, 2019). JAs are oxylipins synthesized from linolenic acid in the chloroplast membranes into 12-oxo-phytodienoic acid (OPDA). Subsequently OPDA is oxidized in the peroxisome to jasmonic acid, which can be further metabolized to various derivatives, including conjugates such as jasmonoyl-l-isoleucine (JA-Ile) which binds to the CORONATINE INSENSITIVE1 (COI1) receptor that triggers the transcriptional regulation of the defense response (Yan *et al.*, 2009). Other phytohormones contribute to shaping the defense response through crosstalk with other hormone signaling pathways, adjusting the plant response to the context perceived (Erb *et al.*, 2012; Berens *et al.*, 2017; Aerts *et al.*, 2021). Among them, ethylene (ET) is a gaseous phytohormone produced from the oxidation of its precursor, 1-aminocyclopropane-1-carboxylate (ACC) by ACC oxidase (ACO) (Yang and Hoffman, 1984). This phytohormone is involved in multiple processes from plant growth, development, and defense, fruit ripening and abscission, senescence, and tolerance, to diverse abiotic stresses (Dubois *et al.*, 2018). It also contributes to the regulation of plant defense and interactions with other organisms through the modulation of JA-dependent responses (Broekgaarden *et al.*, 2015). In Arabidopsis, two main branches in JA-regulated defenses are described, the JA/ET branch, coordinating defenses against necrotrophic pathogens, and the JA/abscisic acid (ABA) coordinated branch, effective against chewing herbivores, and they are believed to be mutually antagonistic (Verhage *et al.*, 2011; Kazan and Manners, 2013; Zhang *et al.*, 2018). Thus, ET is considered antagonist to JA-dependent defenses against herbivory. However, recent findings point to a more complex role for ET, as novel connections between these two pathways have been identified (Ma *et al.*, 2020; Hu *et al.*, 2021; Bungala *et al.*, 2024), but the relevance of JA–ET crosstalk in shaping antiherbivory responses is yet to be uncovered.

Under natural conditions, plants interact simultaneously with multiple organisms, and this leads to a complex fine-tuning of the plant responses, commonly mediated by hormone crosstalk, that shape the final outcome of the multiway interactions (Gruden *et al.*, 2020). Most of the molecular mechanisms that regulate two-way interactions between plants and arthropods are also activated in plant–microbe interactions, but in multiway interactions between plants, microbes, and insects the plant response becomes more complex: additional pathways can be activated, and changes in the intensity and timing of the responses are common (Gruden *et al.*, 2020). In this regard, plant-associated microorganisms, including arbuscular mycorrhizal (AM) fungi, can alter plant responses to attackers above ground, frequently leading to induced plant systemic resistance to diverse pathogens and pests (Pieterse *et al.*, 2014; Pozo *et al.*, 2021).

Over 70% of all vascular plants can establish a mutualistic symbiosis with AM fungi (Brundrett and Tedersoo, 2018). The AM symbiosis confers different benefits to the plant, from better mineral nutrition to better tolerance to biotic and abiotic stresses (Smith and Read, 2008), increasing plant resilience to cope with environmental challenges (Rivero *et al.*, 2018). It can induce resistance to a broad range of pathogens and pests (Jung *et al.*, 2012; Song *et al.*, 2013, 2015; Sanmartín *et al.*, 2020b; Rivero *et al.*, 2021; Fiorilli et al., 2024). The impact on pest feeding and performance may result from changes in the plant nutritional value and chemistry, hormone signaling, and defense responses (Rivero *et al.*, 2021; Dejana *et al.*, 2022; Manresa-Grao *et al.*, 2022; Ramírez-Serrano *et al.*, 2022). Mycorrhiza-induced resistance (MIR) to pathogens and pests commonly relies on the priming of plant defenses (Pozo and Azcón-Aguilar, 2007; Jung *et al.*, 2012). Priming of plant defenses (or immune priming) is a cost-efficient, adaptive defense strategy, in which pre-conditioned tissues are able to activate plant immune responses more efficiently upon challenge—usually leading to faster or stronger defense responses (Conrath *et al.*, 2006, 2015; Martinez-Medina *et al.*, 2016; Mauch-Mani *et al.*, 2017). Different experimental evidence points to a prominent role for JA signaling in the defense priming displayed in mycorrhizal plants (Jung *et al.*, 2012; Song et al., 2013; Mora-Romero *et al.*, 2014; Sanmartín *et al.*, 2020b). However, little is known about the contribution of other hormone signaling pathways to the regulation of mycorrhiza-triggered JA defense priming of MIR (Fiorilli et al., 2024).

Here, we aimed to explore the molecular mechanisms regulating MIR in response to chewing herbivores. We first performed a genome-wide transcriptional profiling of the interactions between tomato, AM fungi, and two different chewing herbivores. Mycorrhizal colonization led to primed JA-regulated responses to the herbivory challenge, but also to a differential regulation of ET signaling before and after herbivory. We found enhanced ET production in mycorrhizal plants, and network analysis pinpointed relevant connections between ET signaling and JA biosynthesis. We hypothesized that ET signaling plays a relevant role in the fine-tuned JA-regulated responses of mycorrhizal plants to herbivory. To test the hypothesis, we used tomato lines impaired in ET synthesis and signaling, and found that the mycorrhiza-associated primed responses were lost in these lines, and they were unable to display MIR against the herbivores. Our results show that differential regulation of ET signaling in mycorrhizal plants is essential for boosting JA production and JA-dependent defenses against both herbivores in tomato, and it is, therefore, a key component of MIR.

## Materials and methods

### Biological material and growing conditions

*Funneliformis mosseae* (T.H. Nicolson & Gerd.) C. Walker & A. Schüßler (BEG12, International Bank of Glomeromycota, https://www.i-beg.eu/cultures/BEG12.htm) is maintained in a pot culture of *Trifolium repens* L. and *Sorghum vulgare* Pers. in a substrate consisting of vermiculite:sepiolite (1:1, v/v) under greenhouse conditions. Tomato seeds were surface-sterilized in 4% sodium hypochlorite for 10 min, washed with water, and incubated in plastic trays containing sterile vermiculite at 25–27 °C, 16/8 h (day/night), and 65–70% relative humidity. After 10 d, seedlings were transplanted into 350 ml pots containing (1:1, v/v) sand:vermiculite mixture. Mycorrhizal treatments consisted of plants inoculated with 10% (v/v) of *F. mosseae* inoculum containing colonized root fragments, spores, and mycelia. Plants were randomly distributed and kept under greenhouse conditions [25–27 °C, 16/8 h (day/night), 65–70% relative humidity]. The fertigation schedule included watering once a week with half-strength Hoagland solution (Hoagland and Arnon, 1938) containing 25% of standard phosphorus concentration. *Spodoptera exigua* Hübner (Lepidoptera: Noctuidae) eggs were obtained from the iDiv (Germany) for experiment 1, and from Universitat de València (Spain) for experiment 2. *Spodoptera exigua* larvae were reared on artificial diet (Hoffman and Lawson, 1964) and maintained at 24 °C. *Manduca sexta* L. (Lepidoptera: Sphingidae) eggs were obtained from iDiv (Germany) for experiment 1, and from Universität Osnabrück (Germany) for experiment 2. Eggs were incubated at 26 °C and larvae were reared on detached tomato leaflets.

### Transcriptional profiling experiment

Surface-sterilized tomato seeds (*Solanum lycopersicum* L. cv. Moneymaker) were used for the transcriptional profiling experiment. The experiment consisted of six treatments: non-mycorrhizal control plants without herbivory (Nm); non-mycorrhizal plants challenged with *S. exigua* (NmSe); non-mycorrhizal plants challenged with *M. sexta* (NmMs); *F. mosseae*-inoculated control plants without herbivory (Fm); *F. mosseae*-inoculated plants challenged with *S. exigua* (FmSe); and *F. mosseae*-inoculated plants challenged with *M. sexta* (FmMs). Each treatment consisted of six independent plants as biological replicates. After 5 weeks, mycorrhizal colonization in roots was evaluated by histochemical staining (*n*=3), and the herbivory assays were performed. Three third instar *S. exigua* larvae or two neonate *M. sexta* larvae were placed on the three apical leaflets of the third true leaf inside a clip cage (30 mm Ø). After 24 h, the larvae were removed, and the leaflets were frozen immediately in liquid nitrogen and stored at −80 °C. The extent of damage was assessed to confirm herbivory, and the damage between herbivores was similar.

### Functional analysis of MIR in ethylene-deficient and insensitive lines

The functional analysis consisted of two independent bioassays, each using distinct herbivore species (*S. exigua* and *M. sexta*) and including two different ET-deficient tomato genotypes lines. Seeds of *S. lycopersicum* wild-type UC82B, ET-deficient line ACD (Klee *et al.*, 1991) and ET-insensitive mutant *Never ripe* (*Nr*) (Wilkinson *et al.*, 1995) were surface-sterilized and germinated as described above. After 8 weeks the herbivory assays were performed on mycorrhizal and non-mycorrhizal plants. Four third instar *S. exigua* larvae or three neonate *M. sexta* larvae were placed on the third fully expanded leaf inside an entomological bag. For the *S. exigua* bioassay, each treatment consisted of seven independent plants, and four larvae were used per plant (a total of 28 larvae per treatment). For the *M. sexta* bioassay, 10 independent plants were used per treatment, and three larvae were used per plant (30 larvae per treatment). Larval mortality and pupation were monitored every 48 h, and *M. sexta* weight was determined at 9 d post-infestation.

### Mycorrhizal quantification

As described in García *et al.* (2020), root samples were cleared and digested in 10% KOH (w/v) for 2 d at room temperature (18–23 °C). Then, root samples were rinsed thoroughly with tap water and acidified with 2% (v/v) acetic acid solution. Fungal root structures were stained with a 5% (v/v) black ink (Lamy, Germany) and 2% acetic acid solution for 24 h at room temperature (Vierheilig *et al.*, 2005). Ink solution was washed with tap water. Mycorrhizal colonization was determined by the gridline intersection method (Giovannetti and Mosse, 1980) using a Nikon SMZ1000 stereomicroscope.

### Ethylene emission quantification by gas chromatography

One detached leaflet of each tomato plant was placed into a 20 ml glass vial containing a sterile filter paper soaked in 200 µl of sterile distilled water to avoid dehydration. The vials were left uncovered for 30 min to avoid the detection of the ET released as a result of the scalpel-induced wounds. After this time, the non-herbivory treatment vials were sealed. For the herbivory treatments, we placed inside the vial one larva of the corresponding herbivore and immediately afterwards the vials were sealed. Vials were maintained at 23 °C under a 18 h photoperiod. A 1 ml aliquot from each vial was withdrawn with a syringe and the area of the ET peak was analyzed in a gas chromatograph with a flame ionization detector (GC-FID; Hewlett Packard 5890). ET emission by the herbivores was determined to be negligible by analyzing vials containing only larvae.

### RNA-seq transcriptional analysis

For RNA-seq analysis, three apical leaflets of the third true leaf contained within the clip cage were harvested and immediately flash-frozen in liquid nitrogen. Three biological replicates per treatment were used, each consisting of pooled material from two plants. Samples were ground in liquid nitrogen. Total RNA was extracted with the RNeasy Plant Mini Kit (Qiagen, Germany) following the manufacturer’s instructions. The quality, quantity, and size of extracted RNA were determined with a Bioanalyzer (Agilent, USA) and Nanodrop (ThermoFisher Scientific, USA). All samples were of good RNA quality (RIN >8, *A*_260_/*A*_280_ >1.8, and *A*_260_/*A*_230_ >2). TruSeq stranded RNA-seq library preparation and paired-end sequencing on the Illumina NovaSeq 6000 platform were performed by Macrogen (South Korea). Quality control of sequencing reads was performed using FastQC (Andrews *et al.*, 2010). Sequences were mapped to the tomato genome version SL4.0 using STAR v2.7.2b (Dobin *et al.*, 2013) and the ITAG 4.0 annotation (Hosmani *et al.*, 2019, Preprint). Differential expression analysis was performed in R using the DESeq2 package v1.26.0 (Love *et al.*, 2014). Prior to statistical testing, genes not having at least 50 counts in at least three samples were excluded. Genes with a false discovery rate (FDR)-adjusted *P*-value <0.05 were considered significantly differentially expressed. Gene set enrichment analysis (GSEA) was performed with TMM-normalized count values using the GSEA tool v4.0.3 (Subramanian *et al.*, 2005) and gene sets based on GoMapMan (Ramšak *et al.*, 2014) BINs. Gene sets with FDR-adjusted *q*-values <0.05 were considered significantly enriched in up- or down-regulated genes. For easier visualization of the enriched gene sets, they were selected and organized in functional supergroups (Supplementary Table S1).

### Network analyses

Network analyses were performed on a specifically generated knowledge network of *S. lycopersicum*. First, the *Arabidopsis thaliana* large comprehensive knowledge network (Ramšak *et al.*, 2018), containing high-quality relations (protein–protein binding, protein–DNA binding, miRNA–transcript targets) between Arabidopsis genes was translated to tomato using PLAZA orthologs (Proost *et al.*, 2015). Additionally, a tomato-specific network of miRNA–transcript targets was generated using psRNATarget (Dai *et al.*, 2018) for tomato miRNAs present in miRBase v22 (Kozomara *et al.*, 2019) and merged with the translated comprehensive knowledge network. A subnetwork with genes that were differentially expressed in at least one of the RNA-seq pipeline contrasts (FDR adjusted *P*-value <=0.05) was extracted in the next step. Shortest path searches were performed using EIN3/EIL1 transcription factors as starting nodes (Solyc01g009170, Solyc01g096810, Solyc06g073720, and Solyc06g073730) and genes related to JA biosynthesis for the end nodes (LOX: Solyc01g099190, Solyc03g006540, Solyc03g122340, Solyc05g014790, Solyc08g014000; AOC: Solyc02g085730; AOS: Solyc04g079730, Solyc10g007960, Solyc11g069800; OPR: Solyc07g007870, Solyc10g086220, Solyc11g013170). Network analyses were performed in R using the igraph package v1.2.8 (Csárdi and Nepusz, 2006), and results were visualized in Cytoscape (Shannon *et al.*, 2003).

### Analysis of gene expression by quantitative reverse transcription–PCR

Total RNA was extracted from leaves using TRIsure™ (Bioline, USA) and treated with DNase I (NZYtech, Portugal). Later, RNA was purified and concentrated using an RNA Clean & Concentrator-5 column kit (Zymo Research, USA). First-strand cDNA was synthesized from 1 µg of purified total RNA using PrimeScript RT Master Mix (TaKara, Japan) according to the manufacturer’s instructions. Quantitative PCRs (qPCRs) and relative quantification of specific mRNA levels were performed with a StepOnePlus™ Real-Time PCR System (Applied Biosystems, USA) and the gene-specific primers described in Supplementary Table S2. Expression values were normalized using the normalizer gene *SlEF-1α* (López-Ráez *et al.*, 2010), encoding the tomato translation elongation factor-1α using the comparative 2^–ΔΔCt^ method (Livak and Schmittgen, 2001). Additionally, the stable expression of *SlEF-1α* compared with other normalizer genes (*SlActin* and *SlCAC*) is shown in Supplementary Figs S1 and S2. For more robust statistical analysis of the differences in expression levels, six independent biological replicates per treatment were analyzed.

### Leucyl aminopeptidase enzymatic activity

Powdered leaf tissue was mixed 1:18 (w:v) with protein extraction buffer [50 mM Tris–HCl (pH 8) and 0.5 mM MnCl_2_]. Samples were centrifuged at 18 000 *g* for 10 min at 4 °C and the supernatant was collected. This process was repeated twice. For the leucyl aminopeptidase (LAP) enzymatic activity, a stock solution of l-leucine-*p*-nitroanilide (LpNA; Sigma-Aldrich, Germany) was prepared in absolute ethanol. The reaction mixture contained 200 µl of 3 mM LpNA [in 50 mM Tris–HCl (pH 8) and 0.5 mM MnCl_2_] and 40 µl of the sample protein supernatant. The reaction was incubated in a 96-well plate at 37 °C for 20 min. The absorbance was measured at 410 nm (Chao *et al.*, 2000).

### Phytohormone analysis

Freeze-dried powdered leaf material was used for hormonal analysis as described by Sánchez-Bel *et al.* (2018) with small changes. A 30 mg aliquot of dry material was homogenized with 1 ml of MeOH:H_2_O with 0.01% of HCOOH containing a pool of a mixture of internal standards of jasmonic-2,4,4d_3_-(acetyl-2,2-d_2_) acid (Sigma‐Aldrich) for both JA and OPDA, and JA‐Ile‐d_6_ synthesized by us for JA-Ile, quantified to a final concentration of 3 µg l^–1^ in the sample. Samples were ground in the cold and centrifuged at 21 000 *g* for 15 min. The pH of the supernatant was reduced to 2.5–2.7 with acetic acid and the extraction was partitioned twice against diethyl ether. The organic phase was recovered and evaporated in a speedvac centrifuge. Samples were resuspended in 1 ml of H_2_O/MeOH (90:10) with 0.01% of HCOOH up to a final concentration of internal standards of 10 ng ml^–1^. The chromatography was performed using a UPLC Kinetex C18 analytical column with a 5 μm particle size, 2.1×100 mm (Phenomenex). Samples were injected onto an Acquity ultraperformance liquid chromatography system (UPLC; Waters, Mildford, MA, USA), which was interfaced with a triple quadrupole mass spectrometer (TSD, Waters, Manchester, UK). Quantification was performed by using Masslynx 4.2 software.

### Statistical analyses

Besides the methods and software for RNA-seq transcriptional analysis described above, statistical analyses were performed with unpaired *t*-test analysis using Statgraphics Plus 3.1. Comparison between treatments of larval mortality and pupation was performed using the log-rank test (Mantel–Cox) with the ‘survival’ and ‘survminer’ packages in R. Principal component analyses (PCAs) were performed using Metaboanalyst software.

## Results

### Mycorrhizal symbiosis impacts transcriptional regulation

**Fig. 1.**
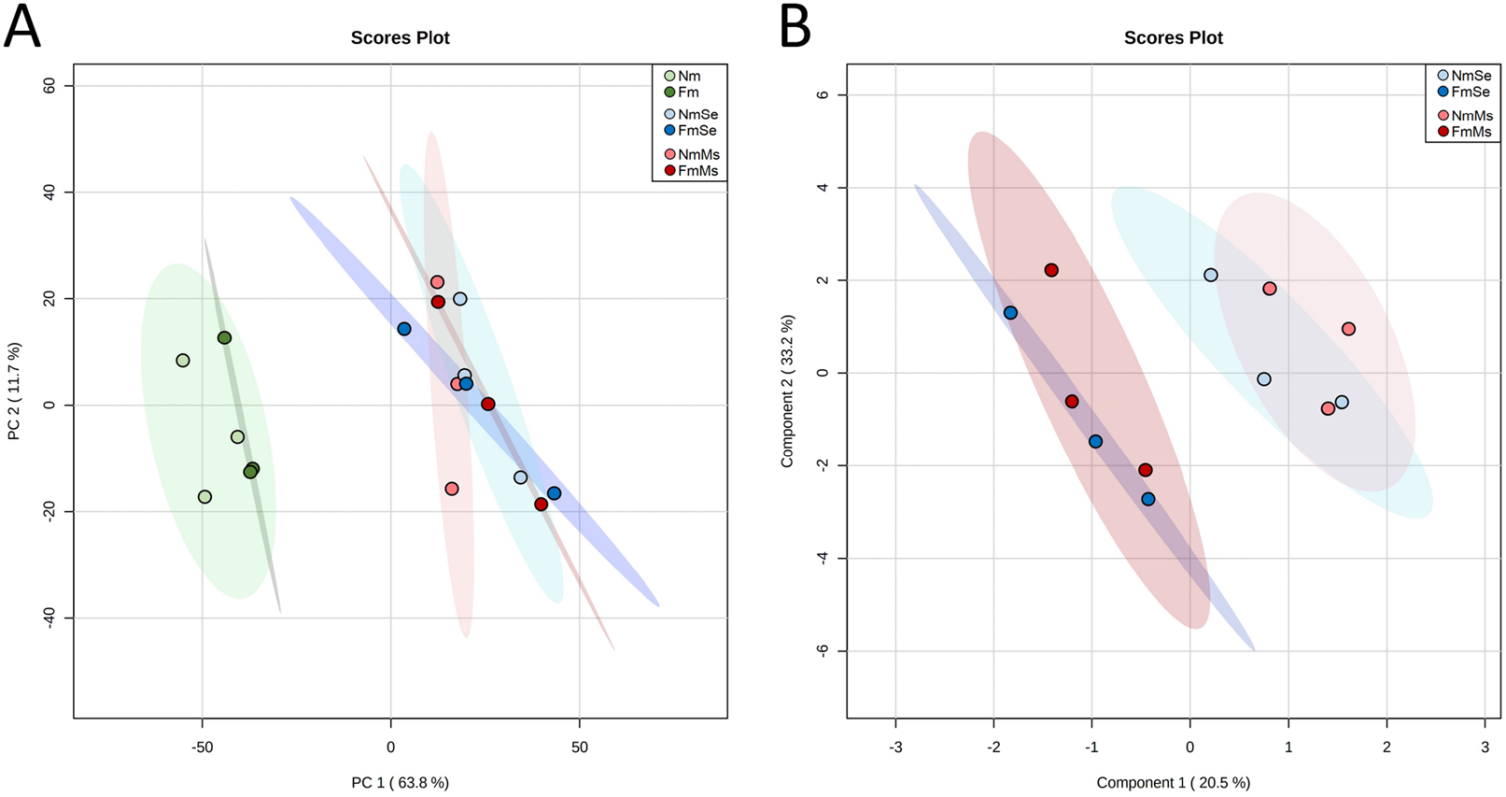
Overview of the impact of the different treatments on the tomato leaf transcriptome. Tomato leaves from uninfested non-mycorrhizal (Nm) and *F. mosseae* mycorrhizal (Fm) plants, or of plants subjected to 24 h of *S. exigua* (NmSe, FmSe) or *M. sexta* (NmMs, FmMs) herbivory. (A) Overall transcriptomic PCA plot for all treatments. (B) Transcriptomic PLS-DA 2-D Scores plot between herbivory treatments only. The percentage of variance explained by the principal component is shown in parentheses.

We previously showed that *F. mosseae* induced resistance in tomato against *S. exigua* by priming the accumulation of antiherbivore compounds (Rivero *et al.*, 2021). To explore the molecular processes underlying the impact of *F. mosseae* on insect performance, we conducted an untargeted transcriptomic study in tomato leaves. Symbiosis establishment was confirmed in the *F. mosseae*-inoculated plants, with mycorrhizal colonization ranging between 15% and 20%. We compared the full transcriptional profile of leaves from non-mycorrhizal (Nm) and mycorrhizal plants colonized by *F. mosseae* (Fm) without challenge, or subjected to herbivory by the generalist *S. exigua* (NmSe and FmSe) and the specialist chewing herbivore *M. sexta* (NmMs and FmMs). PCA was performed on the RNA-seq data (Fig. 1A). The first two principal components explained 75.5% of the total variance. Herbivory had a strong impact on the transcriptome, with a clear separation from non-herbivory treatments in the PCA plot. This separation was mostly explained by PC1, which accounts for 63.8% of the total variance (Fig. 1A). In contrast to the strong effect of herbivory on the leaf transcriptome profile, mycorrhizal colonization itself did not show a significant effect (Nm and Fm; Fig. 1A). However, when focusing only on herbivore-challenged plants, a new multivariate analysis revealed a significant impact of mycorrhization in the transcriptomic profile under herbivory, as illustrated by the separation, mostly explained by PC1, between the non-mycorrhizal (Nm) and mycorrhizal (Fm) plants upon challenge with any of the herbivores (FmSe and FmMs versus NmSe and NmMs; Fig. 1B), pointing to a differential plant response to the herbivore upon mycorrhization.

### Mycorrhizal colonization modulates key regulatory pathways

**Fig. 2.**
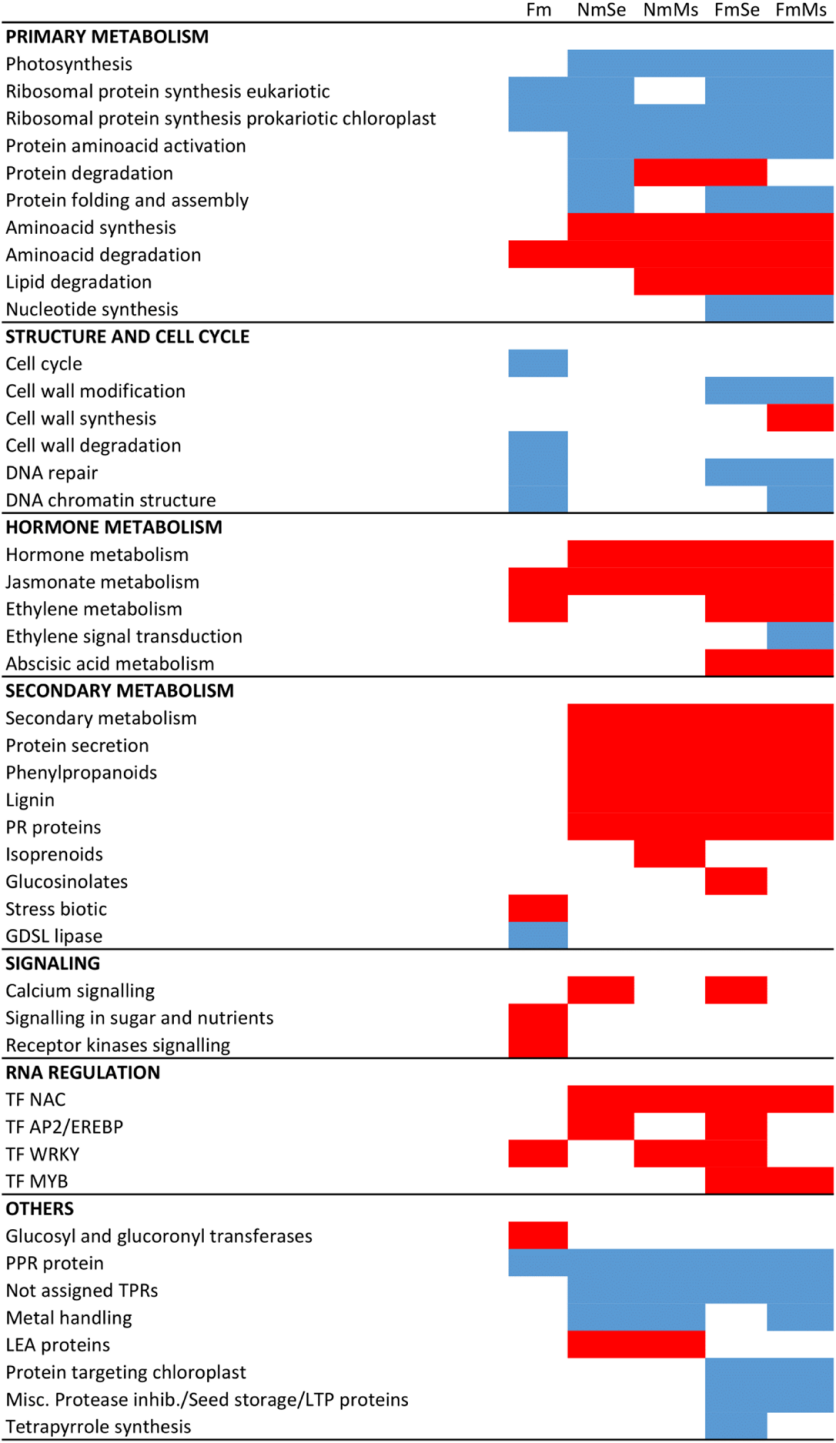
Mycorrhizal colonization impacts plant transcriptional regulation in non-challenged- and herbivory-challenged leaves. Tomato leaves from uninfested non-mycorrhizal (Nm) and *F. mosseae* mycorrhizal (Fm) plants or of plants infested for 24 h with *S. exigua* (NmSe, FmSe) or *M. sexta* (NmMs, FmMs). Heatmap of enriched gene sets in the different treatments as compared with the non-challenged, non-mycorrhizal control (Nm) according to GSEA (FDR <0.05). Blue and red cells indicate repression and induction of the gene set, respectively.

In the absence of herbivory, only 57 differentially expressed genes (DEGs) (FDR <0.05, Supplementary Tables S3–S5) were identified in Fm plants as compared with the Nm controls. We explored the transcriptional changes through GSEA, an analytical method focusing on the regulation of gene groups sharing common biological functions (Subramanian *et al.*, 2005). The GSEA revealed the modulation of several important cell processes in Fm plants (Fm; Fig. 2). Besides changes in cell division and structure (especially in ‘cell cycle’ and ‘cell wall structure’), most changes were found in signaling-related pathways (‘sugar and nutrient signaling’ and ‘receptor kinases signaling’), gene sets related to secondary metabolism (increase in ‘stress biotic receptors’ and ‘UDP glucosyl and glucuronosyl transferases’), and pathways related to plant stress responses (‘DNA chromatin structure’, ‘protein metabolism’, and ‘RNA regulation’). Hormone metabolism was also differentially regulated by *F. mosseae* colonization, as revealed by the enrichment of genes related to the JA and ET pathways (Supplementary Tables S6, S7). It is noteworthy that a high number of transcription factors, particularly related to ET signaling, were observed among the Fm-regulated genes (Supplementary Table S5).

Herbivory had a strong impact on the leaf transcriptome, with 6172 and 4534 DEGs in leaves challenged by the generalist *S. exigua* and the specialist *M. sexta*, respectively (Supplementary Tables S3, S4). When directly comparing plants challenged by the different herbivores (NmSe versus NmMs), no DEGs were found (Supplementary Table S4), suggesting common plant responses to herbivory, probably varying in intensity, with the specialist leading to lower changes in the host. In fact, the GSEA revealed that the general response to both herbivores is conserved, with most functional classes—related to both primary and secondary metabolism—regulated similarly (Fig. 2). Herbivory challenge repressed gene expression related to photosynthesis and impacted synthesis and degradation of amino acids and protein metabolism. Both herbivores activated secondary metabolism, inducing the synthesis of phenylpropanoids and lignins as well as pathogen-related (PR) proteins with antiherbivory functions (mainly proteinase inhibitors, proteases, and polyphenol oxidases) (Fig. 2; Supplementary Table S4). Herbivory also impacted hormone metabolism, mostly by enriching the JA metabolism-related gene set (Fig. 2; Supplementary Table S6). Globally, the impact of the specialist *M. sexta* on primary metabolism was lower that of the generalist *S. exigua*, with a lower repression of photosynthesis- and protein synthesis-related genes (Supplementary Fig. S3).

When focusing on the mycorrhizal effect, we found that the core responses to herbivory were overall similar in mycorrhizal and non-mycorrhizal plants. As in non-mycorrhizal treatments, mycorrhizal plants responded to herbivory with an induced expression of genes related to secondary metabolism and JA-dependent PR proteins involved in defense against chewing herbivores (Fig. 2) and repression of primary metabolism, although this repression was stronger in mycorrhizal than in non-mycorrhizal plants (FmSe and FmMs; Supplementary Fig. S4). Despite the similar responses to herbivory, we found specific differences between herbivory-challenged mycorrhizal and non-mycorrhizal plants related to cell wall synthesis and modification and related to hormone metabolism. While the activation of the JA pathway upon herbivory was common in both Nm and Fm plants, ET and ABA metabolism were up-regulated by herbivory only in mycorrhizal plants (FmSe and FmMs; Fig. 2; Supplementary Tables S6–S8). These results point to a more complex regulation in mycorrhizal plants of hormone pathways in response to herbivory.

### Mycorrhizal symbiosis primes JA-regulated defense responses upon herbivory

**Fig. 3.**
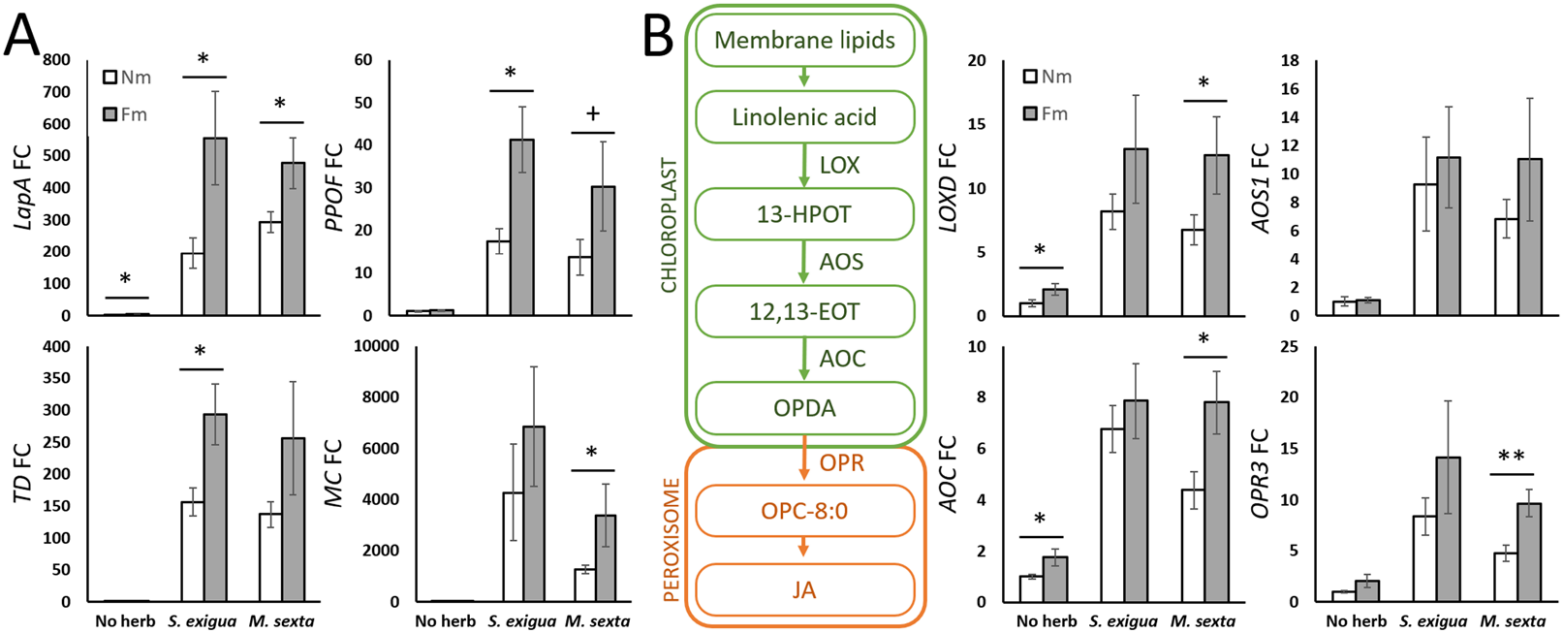
JA-dependent antiherbivory responses and JA biosynthesis upon herbivory are primed in mycorrhizal plants. Tomato leaves from non-mycorrhizal (Nm) and *F. mosseae* mycorrhizal (Fm) plants, uninfested (No herb) or infested for 24 h by *S. exigua* or *M. sexta* (*S. exigua*, *M. sexta*). (A) Relative expression of JA-dependent defense-related marker genes: *leucine aminopeptidase A* (*LapA*, Solyc12g010020), *polyphenol oxidase F* (*PPOF*, Solyc08g074620), *threonine deaminase* (*TD*, Solyc09g008670), and *multicystatin* (*MC*, Solyc00g071180). (B) Relative expression of JA biosynthetic pathway genes: *lipoxygenase D* (*LOXD*, Solyc03g122340), *allene oxide synthase 1* (*AOS1*, Solyc04g079730), *allene oxide cyclase* (*AOC*, Solyc02g085730), and *12-oxophytodienoate reductase 3* (*OPR3*, Solyc07g007870). Expression values were normalized using the reference gene *SlEF*. Data shown are the mean ±SEM of six biological replicates. Statistical analysis was performed with unpaired *t*-test analysis between each herbivory treatment. +*P*<0.1, *P<0.05, **P<0.01.

The transcriptomic analysis confirmed the strong induction of the JA signaling pathway upon herbivory (Supplementary Tables S6, S9). For a more precise analysis of the transcription levels on the JA-regulated antiherbivore defenses, we performed targeted analysis of well-characterized JA-regulated antiherbivory genes: *leucine aminopeptidase A* (*LapA*; Solyc12g010020), *polyphenol oxidase F* (*PPOF*; Solyc08g074620), *threonine deaminase* (*TD*; Solyc09g008670), and *multicystatin* (*MC*; Solyc00g071180). These genes were induced by herbivory in all plants, but the induction was higher in mycorrhizal plants (Fig. 3A), confirming that mycorrhiza lead to primed JA-regulated defenses upon herbivory. We then explored if such changes were related to primed JA biosynthesis by analyzing the expression of JA biosynthetic genes *lipoxygenase D* (*LOXD*; Solyc03g122340), *allene oxide synthase 1* (*AOS1*; Solyc04g079730), *allene oxide cyclase* (*AOC*; Solyc02g085730), and *12-oxophytodienoate reductase 3* (*OPR3*; Solyc07g007870). Remarkably, a small, yet significant induction of the JA biosynthesis genes *LOXD* and *AOC* was detected in unchallenged mycorrhizal plants (Fig. 3B). These changes did not translate into a higher accumulation of the metabolites, as JA, JA-Ile, and OPDA accumulation was similar in mycorrhizal and non-mycorrhizal plants in the absence of herbivory. A robust herbivory-induced accumulation of JAs was found upon herbivory by both caterpillars (Supplementary Fig. S5). The levels were similar in mycorrhizal plants, with only a trend to higher JA and JA-Ile accumulation in *S. exigua*-challenged plants. At the transcriptional level, herbivory induced the expression of JA biosynthesis-related genes in both non-mycorrhizal and mycorrhizal plants, but the induction was overall stronger in mycorrhizal plants. The primed induction was only significant in the case of *M. sexta* infestation (Fig. 3B). The results confirmed boosted transcriptional up-regulation of genes involved in JA synthesis and JA-regulated defenses in herbivory-challenged mycorrhizal plants.

### Mycorrhizal symbiosis enhances ethylene metabolism and primes ethylene biosynthesis and signaling upon herbivory

**Fig. 4.**
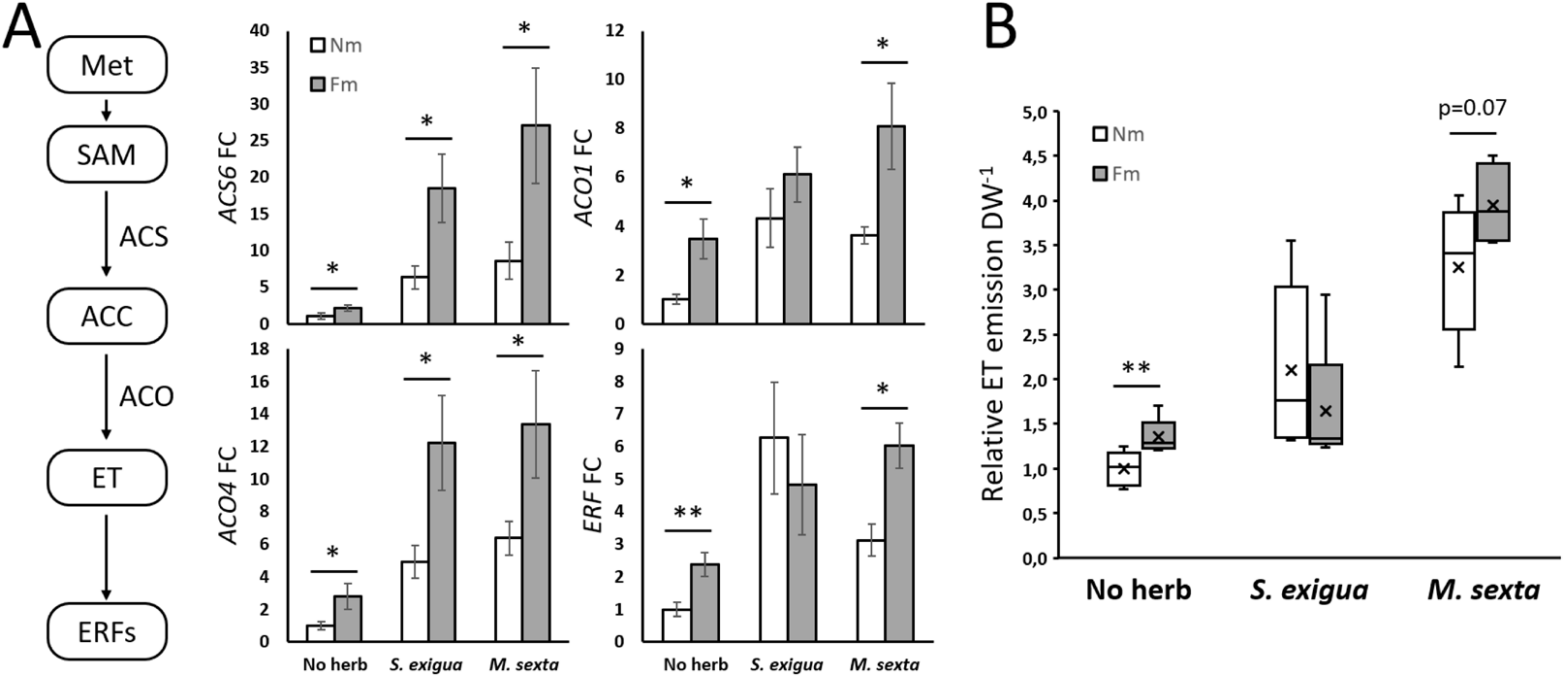
Mycorrhizal symbiosis primes ethylene biosynthesis and signaling. Tomato leaves from non-mycorrhizal (Nm) and *F. mosseae* mycorrhizal (Fm) plants, uninfested (No herb) or infested for 24 h by *S. exigua* or *M. sexta* (*S. exigua*, *M. sexta*). (A) Relative expression of ET biosynthesis genes 1-aminocyclopropane-1-carboxylic acid (ACC) synthase 6 (ACS6, Solyc08g008100), ACC oxidase 1 (ACO1, Solyc07g049530), and ACC oxidase 4-like (ACO4-like, Solyc04g007980), and an ET-responsive factor (ERF, Solyc02g070040). (B) Boxplots show relative ET emission normalized to leaflet DW. Single tomato leaflets of non-mycorrhizal plants (Nm) and mycorrhizal plants with *F. mosseae* (Fm) were challenged with *S. exigua* or *M. sexta* for 18 h inside 20 ml glass vials. A 1 ml aliquot of every sample was withdrawn from the vial and the area of the ethylene peak was analyzed by GC. (A) Expression values were normalized using the reference gene *SlEF*. Data shown are the mean ±SEM of six (A) or five (B) biological replicates. Statistical analysis was performed with unpaired *t*-test analysis between each herbivory treatment. +*P*<0.1, *P<0.05, **P<0.01.

The RNA-seq analysis and GSEA revealed differential regulation of ET-related genes in mycorrhizal plants (Supplementary Tables S5, S7, S10). To more precisely quantify the transcriptional differences related to ET signaling, we performed a targeted analysis of transcriptional regulation of ET biosynthesis and signaling marker genes (Fig. 4A). Higher basal levels in mycorrhizal plants were confirmed for the ET biosynthetic genes *ACC synthase* (*ACS6*; Solyc08g008100) and *ACC oxidase* (*ACO1* and *ACOlike4*; Solyc07g049530 and Solyc04g007980), and for an ethylene-responsive factor (ERF; Solyc02g070040). These genes were also up-regulated in response to both herbivores. Remarkably, as in the case of JA-related genes (Fig. 3), the induction by herbivory of the ET-related genes was generally higher in mycorrhizal plants (Fig. 4A). This primed response was stronger in mycorrhizal plants challenged with *M. sexta* (Fig. 4A), where more JA-regulated mycorrhiza-related changes were also seen (Fig. 3B). We then quantified ET emission (in a new set of plants) to evaluate the relevance of the observed transcriptional up-regulation of ET biosynthesis in mycorrhizal plants. Leaves of *F. mosseae* plants emit significantly more ET than those from non-mycorrhizal plants (Fig. 4B). Herbivory treatments increased ET production in both mycorrhizal and non-mycorrhizal plants, but mycorrhizal plants had slightly higher levels in *M. sexta*-infested leaves (*P*<0.07). The results confirm a differential regulation of ET biosynthesis in mycorrhizal plants.

### Physical interaction networks supports a connection of ethylene signaling with jasmonate biosynthesis

**Fig. 5.**
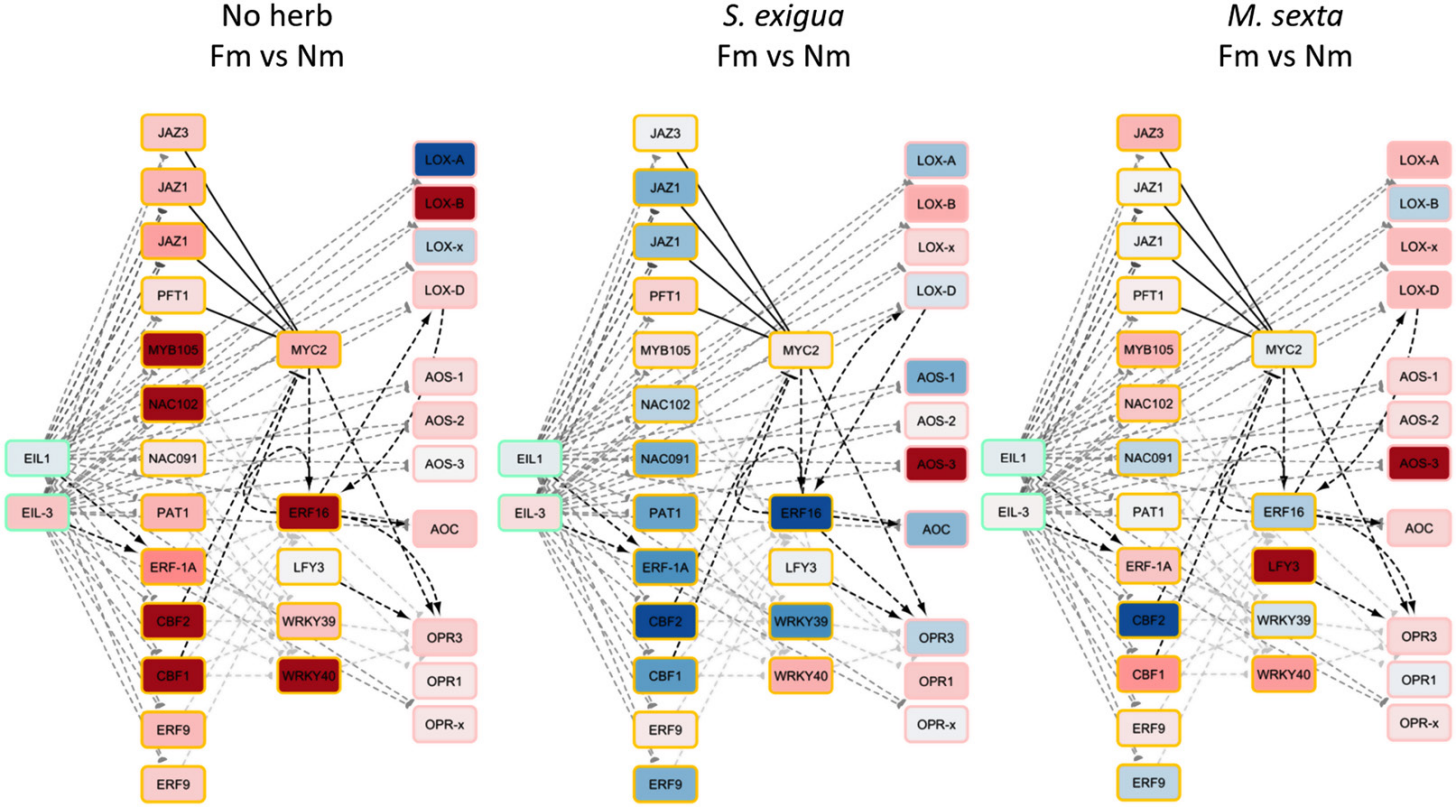
Network analysis visualization for mycorrhizal effects on the JA and ET pathways in response to herbivory. Tomato leaves from non-mycorrhizal (Nm) and *F. mosseae* mycorrhizal (Fm) plants, uninfested (No herb) or infested for 24 h by *S. exigua* or *M. sexta* (*S. exigua*, *M. sexta*). Insight into the network of physical interactions between the signaling network components. Nodes represent tomato protein-coding genes, and the node color shows regulation after the applied treatment, without significance filtering (red=up-regulation, blue=down-regulation). Connection type is shown as different line types (dashed=transcriptional regulation; solid=binding or synthesis); line arrows show the mode of action (arrow=activation; T=inhibition; half-circle=unknown).

To explore the potential interaction between the ET and JA signaling pathways in the differential response of mycorrhizal tomato plants to *S. exigua* and *M. sexta*, the gene expression data were plotted into a physical interaction network, constructed by merging dispersed resources on metabolic pathways, protein–protein interactions, protein–DNA interactions, and small RNA–transcript interactions (Ramšak *et al.*, 2018). We next extracted a subnetwork of genes differentially expressed when comparing leaves of mycorrhizal and non-mycorrhizal plants and their direct interactors. This network was further explored by extraction of the shortest paths between the nodes with a main function in ET signaling (EIN3 and EIN3-like nodes) (left side of the network) and those participating in JA biosynthesis (right side of the network) (Fig. 5). The results show a broad activation of the JA and ET pathway genes in mycorrhizal plants in the absence of herbivory (Fig. 5A). In plants subjected to herbivory (Fig. 5B, C), the symbiosis also had an impact on the two pathways, pointing to an interconnected regulation between both hormones that may lead to the differential regulation of the plant responses to herbivory in mycorrhizal plants. Remarkably, in the *S. exigua* comparison, there are only minimal gene induction changes, reflecting the already high basal activation levels in non-mycorrhizal plants (Fig. 5; Supplementary Table S11). In particular, the expression of *WRKY40* was induced in all three comparisons, with higher levels in mycorrhizal plants regardless of the herbivory status. In addition, ET-responsive factors *ERF16* (ortholog of the Arabidopsis *ORA47*), and *CBF1* and *CBF2*, proposed to act on the JA biosynthetic genes, were differentially regulated in mycorrhizal plants in all three comparisons (Fig. 5). The network results, based on previous experimental data on connections between the different elements, illustrate the interconnected differential regulation of ET and JA signaling elements in mycorrhizal plants. They also pinpoint key regulatory elements potentially mediating the regulatory role of ET on the JA pathway leading to MIR.

### Ethylene signaling is essential for the display of MIR against herbivory

As the network analysis identified potential direct connections of ET signaling with JA biosynthesis and signaling, we hypothesized that ET signaling regulates the boosted JA-related antiherbivore responses triggered by the AM symbiosis. To test this, we performed an experiment using tomato lines impaired in ET synthesis (ACD, expressing the *Pseudomonas* ACC deaminase that cleaves the ET precursor ACC; Klee *et al.*, 1991) or ET perception (*Nr*: *Never ripe*, mutant in the ET receptor ETR3, Wilkinson *et al.*, 1995), both in a UC82B background. Mycorrhizal colonization was well established in all our lines, with no significant differences between the genotypes (Supplementary Fig. S6A). There was no evident effect of the symbiosis on plant biomass in any of the plant genotypes (Supplementary Fig. S6B). We then analyzed ET emission in the different lines. ET was induced upon herbivory in the wild-type, up to 2.5-fold after 3 h of feeding by *M. sexta* larvae (Supplementary Fig. S7). Herbivore-induced ET accumulation was significantly higher in mycorrhizal plants, confirming the primed accumulation of ET in response to herbivory. ET production was almost abolished in the ACD mutant regardless of the treatment and, while ET production still increased in response to herbivory in the ET-insensitive mutant *Nr*, the primed response by mycorrhiza was lost (Supplementary Fig. S7).

**Fig. 6.**
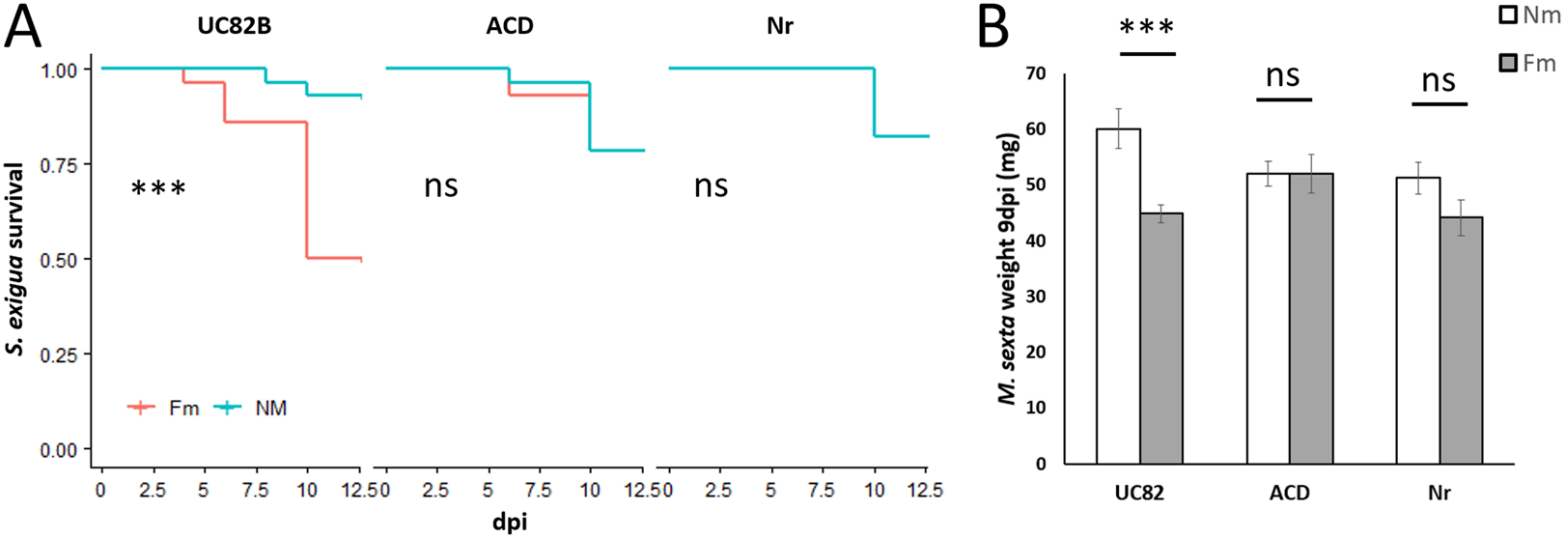
ET-deficient and insensitive lines do not display MIR. (A) Larval survival was monitored every 2–3 d for *S. exigua* larvae and (B) *M. sexta* larval biomass determined 9 d post-infestation (dpi) after feeding on tomato plants of non-mycorrhizal (Nm) and *F. mosseae* mycorrhizal (Fm) plants in the wild-type (UC82B) or ET-deficient and insensitive lines (ACD, Nr). We placed (A) four third instar *S. exigua* larvae or (B) three neonate *M. sexta* larvae on the first true leaf and let them feed inside an entomological bag of (A) seven plants (*n*=28 larvae) and (B) 10 plants (*n*=30 larvae) per treatment. Before they had consumed the whole leaf, we moved them to the next consecutive leaf. Statistical analysis was performed with (A) differences between curves estimated with a log-rank (Mantel–Cox) test and (B) unpaired *t*-test analysis between each genotype. *** *P*<0.001.

We next explored the role of ET in MIR by evaluating MIR in the ET-deficient and insensitive lines. There was a slight, non-significant, reduction in larval survival (Fig. 6A), and a reduction in the total number of pupae in the ET-deficient and insensitive lines as compared with the wild type (Supplementary Fig. S8), which aligns with the traditionally described negative crosstalk between ET- and JA-mediated herbivory defenses (Verhage *et al.*, 2011; Kazan and Manners, 2013; Zhang *et al.*, 2018). This is in agreement with a negative role for ET in basal resistance to herbivory. However, when analyzing the effect of mycorrhizal colonization on plant resistance, we found that in wild-type (UC82B) plants, mycorrhizal colonization significantly increased *S. exigua* larval mortality compared with non-mycorrhizal plants. Notably, this increased mortality and impaired pupation was abolished in both ET-deficient and insensitive lines, ACD and *Nr* (Fig. 6A; Supplementary Fig. S9). Thus, ET plays a positive role in the induced resistance.

Regarding the performance of the specialist *M. sexta*, larval mortality was almost null in all the treatments (Supplementary Fig. S9), with this low mortality probably associated with its high adaptation for *Solanaceae* hosts. Still, larval biomass determination revealed a significant negative effect of the mycorrhizal symbiosis on *M. sexta* performance in the wild type, as the larvae reared on Fm plants had lower biomass compared with those reared on Nm plants (Fig. 6B). This reduction was also lost in the ET-impaired lines (Fig. 6B). Thus, the bioassays with both herbivores demonstrate that ET signaling is required for MIR.

### Ethylene as a positive regulator of MIR-related priming of jasmonate biosynthesis

**Fig. 7.**
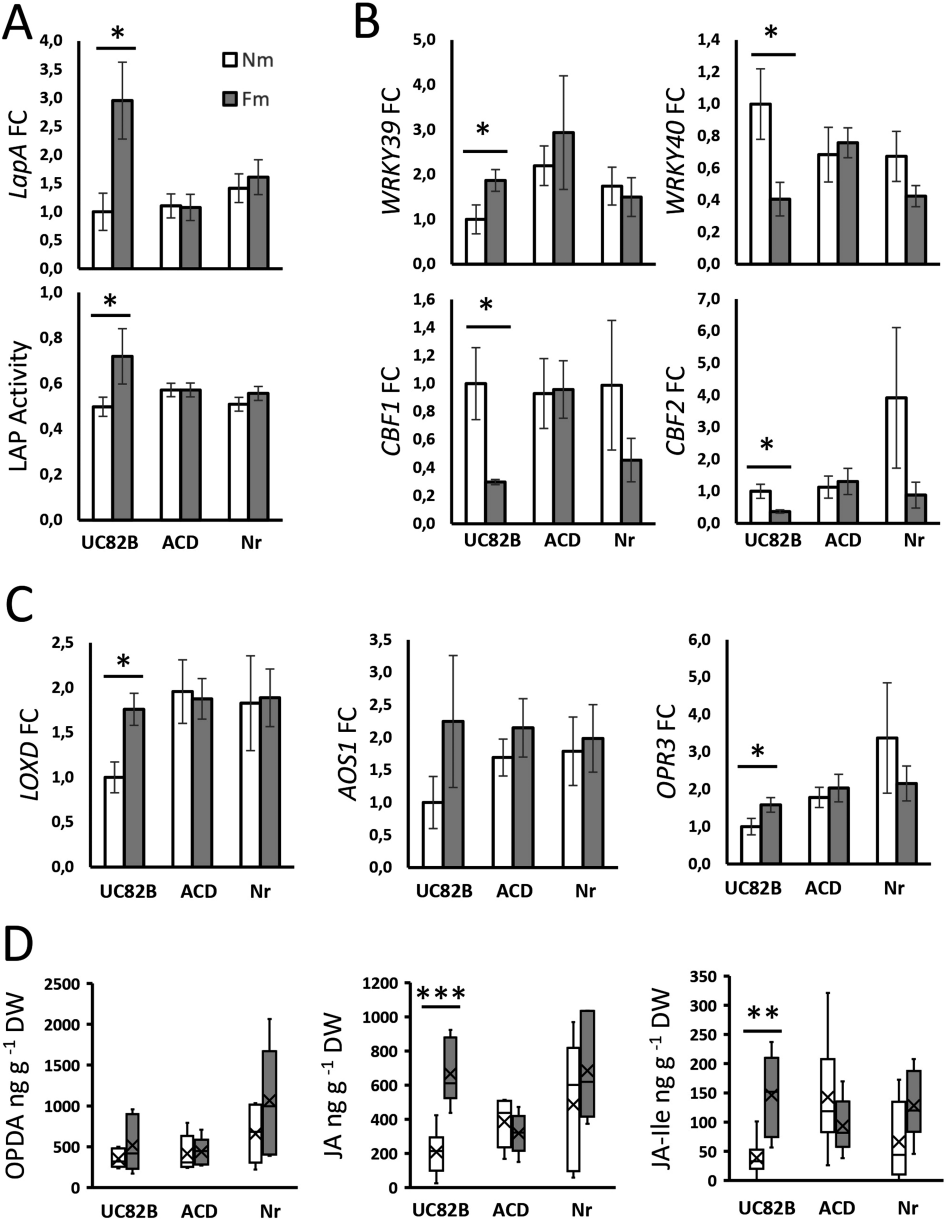
ET is required for the primed JA burst in herbivory-challenged mycorrhizal tomato plants. Tomato plants of non-mycorrhizal (Nm) and *F. mosseae* mycorrhizal (Fm) plants in the wild-type genotype (UC82B) or ET-deficient and insensitive lines (ACD, Nr) subjected to *M. sexta* herbivory. Three larvae were added per plant; newly infested leaves were harvested after 24 h of local herbivory from plants that had been subjected to continuous herbivory for 7 d. (A) Relative expression of the JA-dependent defense-related marker gene *leucine aminopeptidase A* (*LapA*, Solyc12g010020) and the corresponding enzymatic activity (LAP). (B) Relative expression of JA-/ET-related transcription factor genes: *WRKY39* (Solyc03g116890), *WRKY40* (Solyc06g068460), *CBF1* (Solyc03g026280), and *CBF2* (Solyc03g124110). (C) Relative expression of JA biosynthetic pathway genes: *lipoxygenase D* (*LOXD*, Solyc03g122340), *allene oxide synthase 1* (*AOS1*, Solyc04g079730), and *12-oxophytodienoate reductase 3* (*OPR3*, Solyc07g007870). (D) Levels of different oxylipins/JA metabolites (OPDA, JA, and JA-Ile) in the challenged leaves determined by UPLC-MS (A–C). Data represent the mean ±SEM of six biological replicates. Expression values were normalized using the reference gene *SlEF*. (D) Boxplots of six biological replicates normalized to plant DW. Statistical analysis was performed with unpaired *t*-test analysis between each herbivory treatment. *P<0.05, **P<0.01, ***P<0.001.

Finally, to explore the mechanism of MIR regulation by ET, and according to the direct connection between ET and JA signaling indicated by the network analysis (Fig. 5), we evaluated JA-regulated antiherbivory defenses in the ET-deficient and insensitive lines ACD and *Nr*. We focused on the interaction with *M. sexta* because it overall showed the greatest transcriptional changes in mycorrhizal plants as compared with non-mycorrhizal plants (Figs 2–4). We first addressed if the primed JA-regulated defenses to herbivory in mycorrhizal plants require functional ET signaling. In wild-type herbivory-challenged plants, the expression of *LapA*, and the corresponding enzymatic activity, were boosted in mycorrhizal plants compared with non-mycorrhizal plants. This primed response in mycorrhizal plants was completely abolished in both ET-deficient and insensitive lines (Fig. 7A), supporting that ET is required for the priming of JA-regulated defenses associated with MIR.

We next aimed to further disentangle how ET regulates JA-dependent responses during MIR. With this aim, we explored the role of ET signaling in the mycorrhiza-mediated priming of JA biosynthesis. We first analyzed the expression of genes indicated by the network analysis (Fig. 5) as candidates to mediate ET regulation of JA biosynthesis upon herbivory. We evaluated the expression levels of the transcription factor genes *WRKY39* and *WRKY40* (Solyc03g116890 and Solyc06g068460), *LFY3* (Solyc03g118160), and *MYC2* (Solyc08g076930), the APETALA2/Ethylene-response factor genes (AP2/ERF) *ERF16* and *ERF15* (Solyc12g009240 and Solyc06g054630; *ORA47* orthologs), and *CBF1* and *CBF2* (Solyc03g026280 and Solyc03g124110) in the wild-type and the ET-deficient and insensitive lines under herbivory. Differential expression in wild-type mycorrhizal plants was found for *WRKY39*, *WRKY40*, *CBF1*, *CBF2*, and *MYC2* (Fig. 7B; Supplementary Fig. S10). The expression profiles of *WRKY39*, *WRKY40*, and *CBF1* showed consistent differential regulation by mycorrhiza in the RNA-seq and ET functional analysis (Figs 5, 7B) although this differential regulation was in opposite directions. The observed differences may be related to the different plant cultivars and experimental design, including differences in plant and larval age and timing that may lead to differential kinetics in the response. A time-course analysis would help clarify their regulatory dynamics and strengthen our understanding of their roles. Again, these mycorrhiza-associated changes were lost in the ET-deficient and insensitive lines (Fig. 7B; Supplementary Fig. S10). Accordingly, the results confirm the differential, ET-dependent regulation of these genes in mycorrhizal plants. Considering the differential regulation of ABA in mycorrhizal plants under herbivory indicated by the full transcriptome analysis (Fig. 2), we also examined three ABA-dependent genes, two involved in ABA biosynthesis, *NCED1* (Solyc07g056570) and *NCED2* (Solyc01g087260), and a downstream response gene, the dehydrin-coding *Le4* (Solyc02g084850) (Supplementary Fig. S11). *NCED1* and *Le4* showed moderately elevated levels in mycorrhizal wild-type plants, but not in the ACD mutant, but the results are not conclusive, and a more detailed study is required to clarify the role of ABA in MIR. Last, we analyzed the expression of the main JA biosynthetic genes (Fig. 7C). In wild-type challenged plants, mycorrhization led to boosted expression of *LOXD* and *OPR3*, and a similar—although not significant—trend in *AOS1* (Fig. 7C), whereas *AOC* expression was not altered (Supplementary Fig. S10).

Finally, we quantified by UPLC-MS the metabolites OPDA, JA, and JA-Ile, and—in agreement with the increased expression of the biosynthesis genes—we found a higher accumulation of JA and JA-Ile in wild-type challenged mycorrhizal plants, as compared with non-mycorrhizal plants (Fig. 7D). This boosted expression of JA biosynthetic genes and JA accumulation triggered by mycorrhiza was completely lost in the ET impaired lines (Fig. 7C, D). Our results confirm that ET is required for the mycorrhizal priming of JA biosynthesis in herbivory-challenged plants leading to MIR.

## Discussion

MIR has been described in different plant species, mostly against necrotrophic pathogens and leaf-chewing insects (Hartley and Gange, 2008; Roger *et al.*, 2013; Song *et al.*, 2013; Nair *et al.*, 2015; Sanchez-Bel *et al.*, 2016; He *et al.*, 2017; Sanmartín *et al.*, 2020a, b; Rivero *et al.*, 2021). The susceptibility of these aggressors to JA-regulated plant defense responses suggested the involvement of JA signaling in MIR (Pozo and Azcón-Aguilar, 2007). This hypothesis was supported by results showing transcriptional regulation of JA-dependent defenses associated with MIR, and the use of JA-deficient lines confirmed the central role of JA as a main regulator of MIR (Song *et al.*, 2013; Mora-Romero *et al.*, 2014). Further studies including omics approaches illustrated the complexity of MIR-associated responses, suggesting the existence of additional regulatory elements in the process (Sanmartín *et al.*, 2020b; Rivero *et al.*, 2021), but the molecular regulation of defense priming in MIR remains undiscovered. Here we followed an untargeted approach to identify novel elements in the regulation of MIR against chewing herbivores in tomato. Our analysis revealed primed JA-regulated antiherbivore responses in mycorrhizal plants, and pinpointed ET as a potential key regulatory element of the primed response. By combining transcriptomic, enzymatic, and metabolomic analyses, herbivore bioassays, and a genetic approach using ET-deficient and insensitive lines, we were able to demonstrate that ET signaling plays a central role in MIR.

Mycorrhizal colonization has a strong impact on root transcriptome and metabolome profiles, as shown in different plants including tomato (López-Ráez *et al.*, 2010; Rivero *et al.*, 2015, 2018; Vangelisti *et al.*, 2018; Hsieh *et al.*, 2022; Jing *et al.*, 2022), but usually generates only minor changes in the leaves in the absence of challenge (Song et al., 2013; Schweiger *et al.*, 2014; Adolfsson *et al.*, 2017; Rivero *et al.*, 2021). In our full transcriptome analysis (Fig. 1), we also found a low number of DEGs in mycorrhizal plants in the absence of challenge, but more differences were observed upon herbivory, fitting with a primed plant response to the aggressor. Nevertheless, while few significant DEGs were identified in mycorrhizal plants in the absence of challenge, GESAs revealed that the symbiosis impacts diverse functional categories that could contribute to their primed state (Fig. 2). For example, mycorrhizal symbiosis triggered changes related to transcriptional regulation, in histone and chromatin compaction, as well as in the abundance of transcription factors and receptor kinases. These elements have been proposed to underlie the primed state in pre-conditioned plants (Conrath *et al.*, 2015). In addition, the categories corresponding to JA and ET metabolism were also up-regulated by mycorrhiza at the basal level, pointing to differential hormone homeostasis in these plants. All these changes are consistent with the premise that defensive priming generates few basal changes in the organism, but in key regulatory aspects, that may allow a boosted response upon challenge (Martinez-Medina *et al.*, 2016; Mauch-Mani *et al.*, 2017). Indeed, upon herbivory, differences between mycorrhizal and non-mycorrhizal plants were more pronounced, supporting a primed response to the attack.

Herbivory led to a strong transcriptional reprogramming in the plant, regardless of its mycorrhizal status (Fig. 2). The transcriptomic analysis evidenced a core of common plant responses to both herbivores, including the up-regulation of plant secondary metabolism for defense, and the reorganization of primary metabolism. Nevertheless, we observed some variations in the response to the herbivores, as some categories were altered in the interaction with the generalist *S. exigua* but not with the specialist *M. sexta*, which could be related to their degree of specialization (Ali and Agrawal, 2012), as specialists try to circumvent plant defense activation. Certainly, both herbivores activated the JA signaling pathway, as it is the central pathway of resistance against chewing herbivores (Wasternack and Hause, 2013; Erb and Reymond, 2019).

While these responses to herbivory were overall common in non-mycorrhizal and mycorrhizal plants, more changes were detected in mycorrhizal plants, with some exclusive changes in categories related to hormone metabolism, cell wall modifications, transcription factors, and protease inhibitors (Fig. 2). In particular, the induction of JA-regulated responses was boosted in mycorrhizal plants, as they displayed a stronger transcriptional activation of antiherbivory defenses (Fig. 3), for example of genes coding for the well-characterized defensive LAPA, MC, TD, and PPOs (Green and Ryan, 1972; Felton *et al.*, 1989; Chen *et al.*, 2005; Erb and Reymond, 2019).

Remarkably, besides the activation of JA responses, ABA and ET pathways were also differentially regulated in mycorrhizal plants (Fig. 2). Such changes in additional signaling pathways could contribute to a more complex regulation of antiherbivore responses. Indeed, hormone crosstalk is key in shaping final defense responses in plants (Erb *et al.*, 2012; Berens *et al.*, 2017; Aerts *et al.*, 2021). ET is an essential regulator of plant defenses, playing a complex modulatory role in the overall hormonal crosstalk shaping plant defense responses to specific challenges (Broekgaarden *et al.*, 2015). While it is well documented that herbivory damage induces ET emission in plants (Winz and Baldwin, 2001; de Vos *et al.*, 2005), ET signaling is believed to play a negative role in resistance to herbivores. For example, in Arabidopsis, the ABA/JA synergy orchestrates an effective defense response to chewing insects, that is fine-tuned through antagonism with the ET-regulated pathway (Bodenhausen and Reymond, 2007; Verhage *et al.*, 2011). Antagonism between ET and ABA/JA pathways also impairs nicotine synthesis against *M. sexta* larvae in *Nicotiana attenuata*, resulting in diversified defensive responses (von Dahl and Baldwin, 2007; Onkokesung *et al.*, 2010). Not surprisingly, attackers can modulate ET signaling to alter hormone crosstalk for their benefit (Vogel *et al.*, 2007) and, for example, elicitors in *S. exigua* saliva manipulate ET signaling to suppress JA responses in *Medicago* (Paudel and Bede, 2015). On the other hand, ET/JA signaling is required for phenolamide accumulation in response to herbivory (Li *et al.*, 2018, 2020; Wang *et al.*, 2020; Figon *et al.*, 2021), and recent studies point to the potential synergy of JA and ET also in resistance to herbivory (Hu *et al.*, 2021). Thus, experimental evidence illustrates the complex regulatory role of ET in shaping defensive responses, probably fine-tuning defenses through positive and negative interactions with other pathways depending on timing and hormone doses. Our data suggest that ABA may also contribute to shaping the plant defense responses during MIR (Fig. 2), but in-depth analysis including functional analysis with ABA-deficient mutants is required to evaluate its precise contribution to defense priming.

In this study we focused on the potential role of ET signaling in the defense priming associated with MIR. Our analyses revealed that mycorrhizal symbiosis enhanced ET synthesis in leaves under basal conditions, and these increased ET levels are further amplified upon herbivory (Fig. 4). We hypothesized that this differential regulation of the ET pathway in mycorrhizal plants is essential for MIR against herbivory. The relevance of JA–ET interplay in microbe-induced plant resistance has been reported against necrotrophic pathogens, mostly in the Arabidopsis–*Pseudomonas* WCS417r model system (van Loon *et al.*, 2006; Pieterse *et al.*, 2014). However, the involvement of ET in induced resistance against herbivores has been only proposed in the Arabidopsis–*Pseudomonas* WCS417r-induced resistance against *Mamestra brassicae* (Pangesti *et al.* 2016). Here, we used two ET-deficient lines to perform functional analysis to address the contribution of ET signaling to MIR. ET is known to be a negative regulator of the mycorrhizal symbiosis and, accordingly, the ET overexpressor mutant *epinastic* showed reduced mycorrhization levels (de Los Santos *et al.*, 2011; Fracetto *et al.*, 2013, 2017). However, the ET-deficient ACD (impaired in ET production) and the ET-insensitive *Nr* (deficient in ET signaling) used here showed functional mycorrhizal establishment, with colonization levels similar to the wild-type line. Previous studies also showed only minor and transient differences in the mycorrhizal colonization of the *Nr* mutant, in contrast to the marked reduction in *epinastic* (Fracetto *et al.*, 2013). Despite the similar mycorrhizal colonization levels among the lines, both mutants were unable to display MIR against the two herbivores tested, supporting the key role of ET in the enhanced resistance (Fig. 6). Regarding plant defense responses, wild-type mycorrhizal plants showed primed expression and activity levels of LapA upon herbivory. LapA is considered a marker of JA-regulated antiherbivore defenses but, in addition, it acts as a chaperone regulating late JA and wound responses (Fowler *et al.*, 2009). Accordingly, the changes in LapA levels may have further consequences on the plant defensive response through differential post-transcriptional modifications. Remarkably, the primed LapA activity in mycorrhizal plants, and the enhanced resistance to the herbivores, was lost in the ET-deficient and insensitive mutants, confirming that ET is required for mycorrhizal priming of JA-regulated antiherbivore defenses and MIR.

Finally, the results of the transcriptome and network analyses suggested that the ET effect may act upstream of JA signaling, probably targeting JA biosynthesis. The effect of ET on JA biosynthesis in response to wounding was only studied to a moderate degree earlier. Previous studies demonstrated that inhibition of ET signaling partially inhibited JA synthesis (O’Donnell *et al.* 1996), and ORA47, an AP2/ERF, was shown to positively regulate OPDA biosynthesis by binding to the promoters of most JA biosynthetic genes (Pauwels *et al.*, 2008; Chen *et al.*, 2016; Hickman *et al.*, 2017). Thus, considering the described antagonism and synergism between ET and JA, the evidence suggests a modulatory role for ET that combines both positive and negative effects on the JA pathway. Timing in the sequential induction of phytohormones seems to be essential for the crosstalk outcome: in late stages of herbivory attack, ET may be antagonistic to JA defenses, but ET seems to be required for the JA burst upon challenge.

Our network analysis pointed to some regulatory elements as candidates to mediate the positive impact of ET signaling on JA biosynthesis (Fig. 5), and differential regulation in mycorrhizal plants was confirmed for the ET-responsive factor genes *WRKY39*, *WRKY40*, *CBF1*, and *CBF2*. The follow-up analyses on the ET-deficient and insensitive lines revealed that their differential regulation, the boosted expression of the JA biosynthesis genes *LOXD* and *OPR3*, as well as the enhanced accumulation of JA and its bioactive conjugate JA-Ile observed in mycorrhizal wild-type plants was lost in these lines (Fig. 7). Thus, our study demonstrates that the regulation of ET signaling in mycorrhizal plants is required for the primed accumulation of JA upon herbivory in mycorrhizal plants. The results agree with recent studies showing that, in tomato, *ORA47* orthologs *ERF15* and *ERF16* are responsible for the quick transcriptional activation of JA synthesis upon herbivory by inducing *LOXD*, *AOC*, and *OPR3* transcription (Hu *et al.*, 2021). Whether this differential ET production/function is required in roots and/or shoots deserves further investigation including grafting experiments with wild-type and ET-deficient tomato genotypes.

In conclusion, our study provides comprehensive evidence that ET signaling plays a critical role in the primed defense responses that lead to enhanced resistance in mycorrhizal plants. The differential regulation of ET biosynthesis in mycorrhizal plants leads to a boosted JA burst upon herbivore attack, and the primed activation of downstream JA-dependent responses. Our results contribute to elucidating the complex hormone crosstalk in shaping plant defense priming in mycorrhizal plants, with ET acting as a pivotal regulator of JA defenses upon herbivory. Understanding the regulation of plant defense priming by beneficial microbes paves the way for improving biotechnological applications of microbial inoculants in sustainable crop protection.

## Supplementary data

The following supplementary data are available at *JXB* online.

Fig. S1. Additional information on gene expression normalization for the transcriptional profiling experiment.

Fig. S2. Additional information on gene expression normalization for the experiment involving ethylene-deficient and insensitive lines.

Fig. S3. Heatmap of *S. exigua* treatment (NmSe) changes on enriched gene sets compared with *M. sexta* treatment (NmMs).

Fig. S4. Heatmap of mycorrhizal herbivory treatment changes on enriched gene sets compared with their non-mycorrhizal herbivory controls.

Fig. S5. Levels of oxylipins/JA metabolites in tomato leaves in non-challenged and herbivory-challenged non-mycorrhizal and mycorrhizal plants.

Fig. S6. Mycorrhizal root colonization and shoot biomass of wild-type and ET-deficient and insensitive lines.

Fig. S7. Relative ET emission of non-mycorrhizal and mycorrhizal wild-type and ET-deficient and insensitive lines.

Fig. S8. *S. exigua* pupation in non-mycorrhizal and mycorrhizal wild-type and ET-deficient and insensitive lines.

Fig. S9. *M. sexta* mortality in non-mycorrhizal and mycorrhizal wild-type and ET-deficient and insensitive lines.

Fig. S10. Relative expression of JA/ET-related transcription factor genes in wild-type and ET-deficient and insensitive lines after 24 h of *M. sexta* herbivory.

Fig. S11. Relative expression of ABA-dependent genes in wild-type and ET-deficient and insensitive tomato lines after 24 h of *M. sexta* herbivory.

Table S1. GSEA manually organized functional supergroups from enriched gene sets.

Table S2. Primers used for qPCR.

Table S3. Overview of RNA-seq DEGs.

Table S4. Total processed RNA-seq.

Table S5. RNA-seq of Fm versus Nm DEGs.

Table S6. RNA-seq of the JA pathway.

Table S7. RNA-seq of the ET pathway.

Table S8. RNA-seq of the ABA pathway.

Table S9. RNA-seq read counts for JA.

Table S10. RNA-seq read counts for ET.

Table S11. Network genes.

eraf053_suppl_Supplementary_Figures_S1-S11_Tables_S1-S3

eraf053_suppl_Supplementary_Table_S4

eraf053_suppl_Supplementary_Table_S5

eraf053_suppl_Supplementary_Table_S6-S8

eraf053_suppl_Supplementary_Table_S9-S10

eraf053_suppl_Supplementary_Table_S11

## Data Availability

RNA-seq data are openly available at the National Center for Biotechnology Information (NCBI) Sequence Read Archive (SRA) (https://www.ncbi.nlm.nih.gov/sra) under the SRA accession SRP458983. The raw data that support the findings of this study are available from the corresponding author upon request.
