# Supplementary material for: Ethylene signaling is essential for mycorrhiza-induced resistance against chewing herbivores in tomato
Source: J Exp Bot. 2025 Feb 8;76(7):2005–21. doi: 10.1093/jxb/eraf053 (PMC12066123; doi:10.1093/jxb/eraf053)
Supplement: eraf053_suppl_Supplementary_Figures_S1-S11_Tables_S1-S3 [file eraf053_suppl_supplementary_figures_s1-s11_tables_s1-s3.pdf]

Supplemental Fig. S1. Additional information on gene expression normalization for the transcriptional profiling experiment.

Supplemental Fig. S2. Additional information on gene expression normalization for the ethylene deficient and insensitive lines experiment.

Supplemental Fig. S3. Heatmap of *S. exigua* treatment (NmSe) changes on enriched gene sets compared with *M. sexta* treatment (NmMs)

Supplemental Fig. S4. Heatmap of mycorrhizal herbivory treatments changes on enriched gene sets compared with their non-mycorrhizal herbivory controls

Supplemental Fig. S5. Levels of oxylipins/JA metabolites in tomato leaves in non-challenged and herbivory-challenged from non-mycorrhizal and mycorrhizal plants.

Supplemental Fig. S6. Mycorrhizal root colonization and shoot biomass of wt and ET-deficient and insensitive lines

Supplemental Fig. S7. Relative ET emission of non-mycorrhizal and mycorrhizal wt and ET-deficient and insensitive lines

Supplemental Fig. S8. *S. exigua* pupation in non-mycorrhizal and mycorrhizal wt and ET-deficient and insensitive lines

Supplemental Fig. S9. *M. sexta* mortality in non-mycorrhizal and mycorrhizal wt and ET-deficient and insensitive lines

Supplemental Fig. S10. Relative expression of JA-ET related transcription factor genes in wt and ET-deficient and insensitive lines after 24h of *M. sexta* herbivory.

Supplemental Fig. S11. Relative expression of ABA-dependent genes in wt or ET deficient and insensitive tomato lines after 24h of *M. sexta* herbivory

Supplemental Table S1. GSEA Manually organized functional supergroups from enriched gene sets

Supplemental Table S2. Primers used for qPCR

Supplemental Table S3. RNA-Seq DEGs overview

ID	Solyc ID	protein	Read counts NM	Fm	Se	Fm_Se	Ms	Fm_Ms
**GAPDH**	Solyc05g014470	glyceraldehyde 3-phosphate dehydrogenase	158370	144117	110491	137965	132789	120770
**TBP**	Solyc01g028930	TATA-box-binding protein (AHRD V3.3 *** A0A1U8HIY4_CAPAN)	2205	2308	2077	2457	2155	2155
**EF1α**	Solyc06g005060	elongation factor 1-alpha	106784	99354	84035	97068	98191	89209
**RPL8**	Solyc10g006580	ribosomal protein L2	25612	23391	18530	21191	22777	19641
**DNAJ**	Solyc04g009770	DnaJ protein (AHRD V3.3 *** Q43177_SOLTU)	39213	41570	34560	39603	39562	35328
**TIP41**	Solyc10g049850	TIP41-like protein (AHRD V3.3 *** A0A200QZN1_9MAGN)	4044	4204	3730	4879	4334	4129
**SAND**	Solyc03g115810	Vacuolar fusion protein mon1	5666	5338	5514	6652	5632	5910
**CAC**	Solyc08g006960	AP-2 complex subunit mu (AHRD V3.3 *** A0A2G2W3I9_CAPBA)	4465	4552	4292	5479	4701	5089
**Expressed**	Solyc07g025390	dimethylallyl adenosine tRNA methylthiotransferase (AHRD V3.3 *** AT4G33380.1)	995	942	843	1021	938	903
**Actin**	Solyc11g005330	Actin	55789	54971	45045	56362	52204	53432

Supplemental Fig. S1. Stability of the *SlEF-1α* normalization compared to other normalizer genes for gene expression of the transcriptional profiling experiment. Data shown are read counts of different normalizer genes obtained from the RNA-Seq analysis.

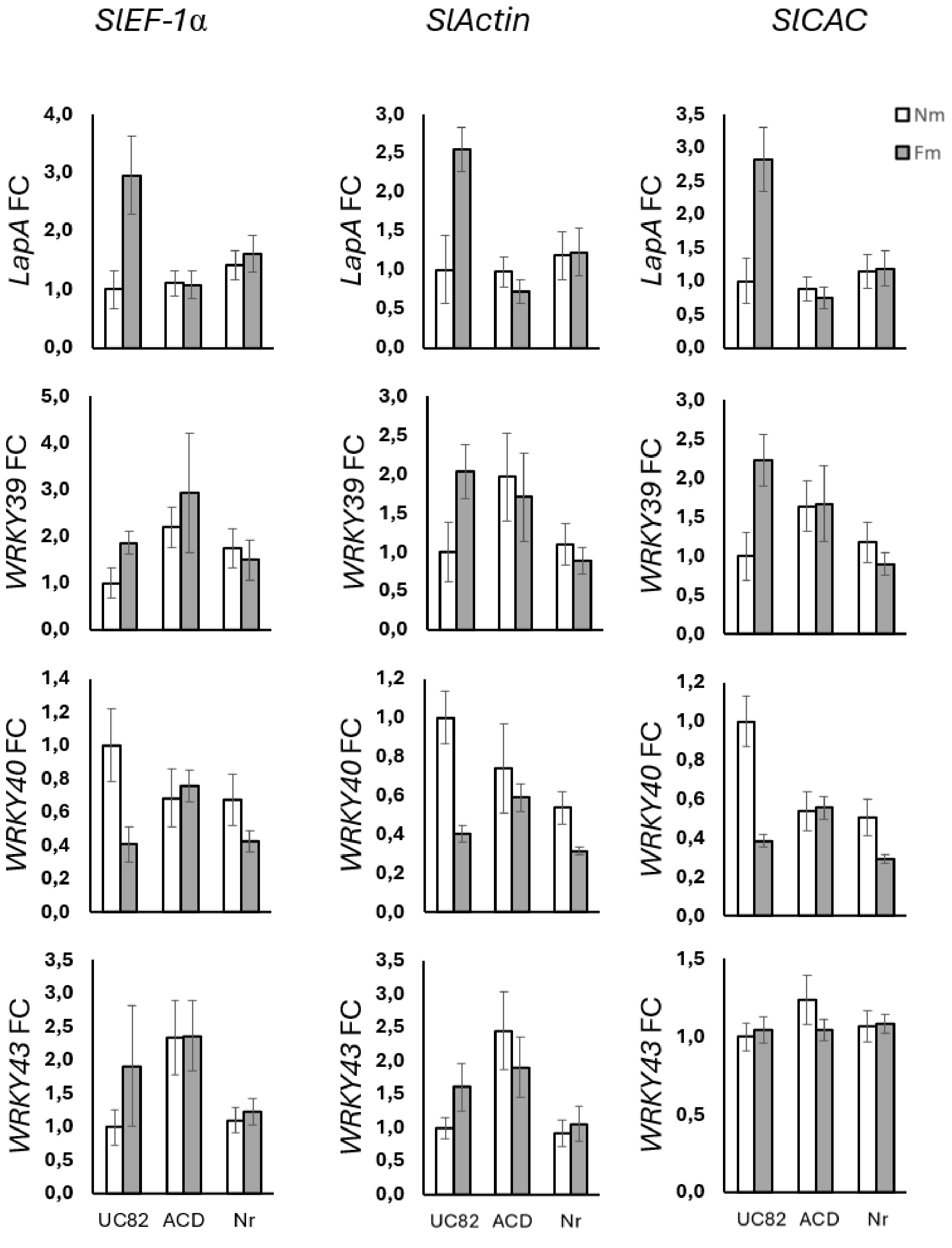

Supplemental Fig. S2. Stability of the *SlEF-1α* normalization compared to other normalizer genes for gene expression the ethylene deficient and insensitive lines experiment. Expression values were normalized using the normalizer genes *SlEF-1α* (Solyc06g009960), *SlActin* (Solyc11g005330), and *SlCAC* (Solyc08g006960).

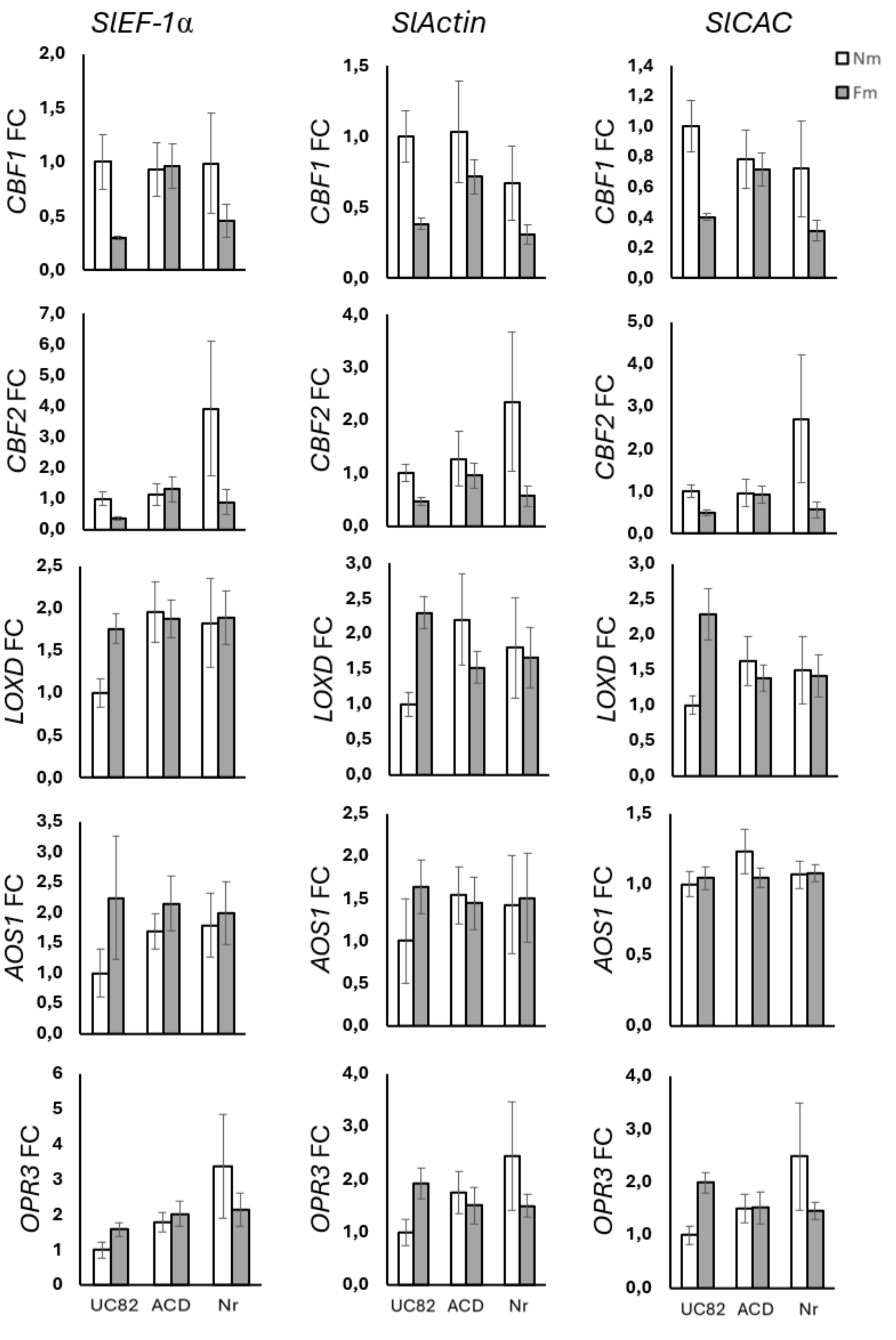

Supplemental Fig. S2. *Continued* Stability of the *SlEF-1α* normalization compared to other normalizer genes for gene expression the ethylene deficient and insensitive lines experiment. Expression values were normalized using the normalizer genes *SlEF-1α* (Solyc06g009960), *SlActin* (Solyc11g005330), and *SlCAC* (Solyc08g006960).

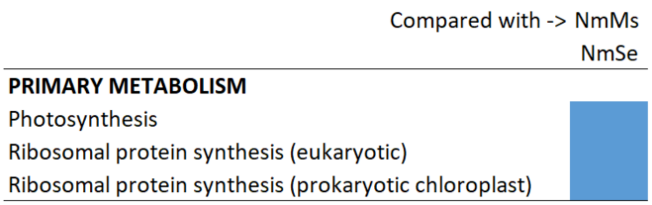

Supplemental Fig. S3. *M. sexta* has a lower impact on the primary metabolism compared with *S. exigua*. Tomato leaves from uninfested non-mycorrhizal (Nm) and *F. mosseae* mycorrhizal (Fm) plants or subjected to 24 h of *S. exigua* (NmSe, FmSe) or to *M. sexta* (NmMs, FmMs) herbivory. Heatmap of *S. exigua* treatment (NmSe) changes on enriched gene sets compared with *M. sexta* treatment (NmMs) according to GSEA results of 3 biological replicates, each consisting of a pool of two plants (FDR<0.05). Blue and red cells indicate repression and induction of the gene set, respectively.

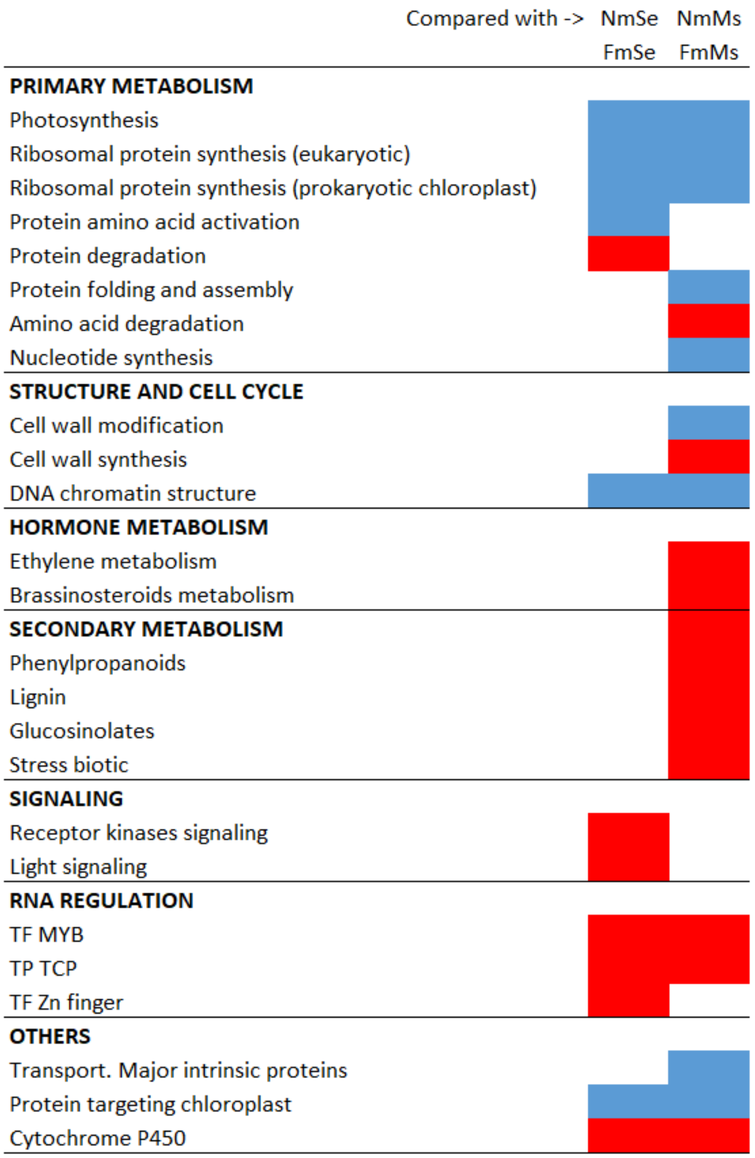

Supplemental Fig. S4. Mycorrhizal colonization deepens the primary metabolism repression upon herbivory and upon *M. sexta* boosts the secondary metabolism. Tomato leaves from non-mycorrhizal (Nm) and *F. mosseae* mycorrhizal (Fm) plants were subjected to 24 h of *S. exigua* (NmSe, FmSe) or to *M. sexta* (NmMs, FmMs) herbivory. Heatmap of enriched gene sets of the different mycorrhizal herbivory treatments as compared with their corresponding non-mycorrhizal treatment, according to GSEA results of 3 biological replicates, each consisting of a pool of two plants (FDR<0.05). Blue and red cells indicate repression and induction of the gene set, respectively.

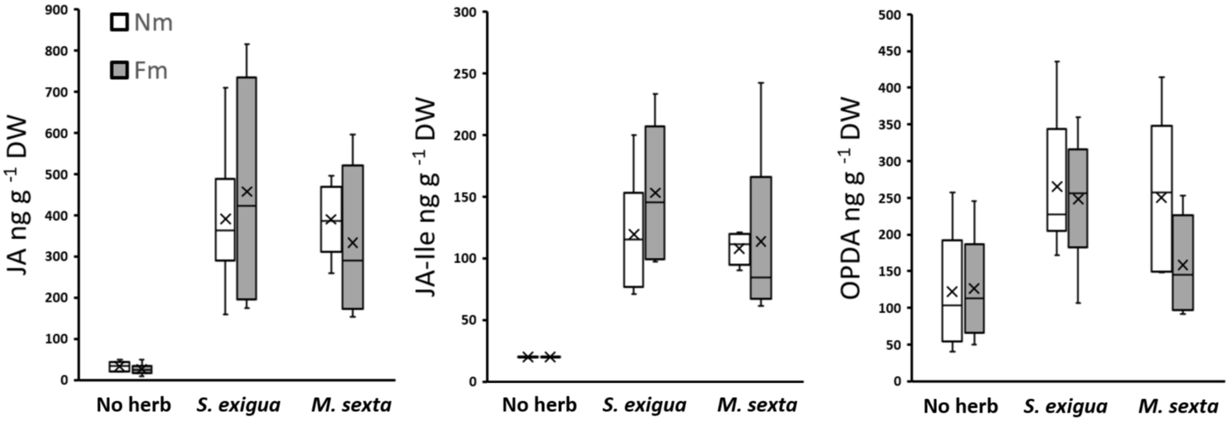

Supplemental Fig. S5. Levels of oxylipins/JA metabolites in tomato leaves in non-challenged and herbivory-challenged from non-mycorrhizal and mycorrhizal plants. Tomato leaves from non-mycorrhizal (Nm) and *F. mosseae* mycorrhizal (Fm) plants, uninfested (No herb) or infested for 24 h by *S. exigua* or *M. sexta* (*S. exigua*, *M. sexta*). JA, JA-Ile and OPDA levels determined by UPLC-MS. Boxplots of 6 biological replicates normalized to plant dry weight (DW). Statistical analysis was performed with unpaired t-test analysis between each herbivory treatment.

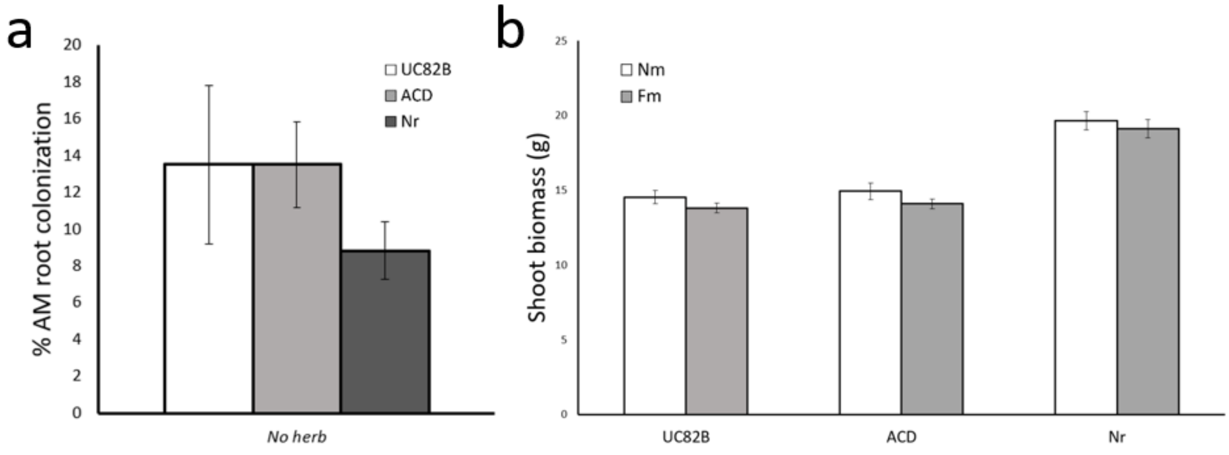

Supplemental Fig. S6. ET-deficient and insensitive lines showed no differences in (a) AM colonization nor (b) mycorrhizal effects on shoot biomass. Tomato plants of non-mycorrhizal (Nm) and *F. mosseae* mycorrhizal (Fm) plants in the wild-type (UC82B) or ET-deficient and insensitive lines (ACD, Nr) without herbivory (No herb). Mycorrhizal root colonization and shoot biomass was determined 8 weeks post inoculation. Data shown as mean ± SEM of (a) 6 or (b) 10 biological replicates. Statistical analysis was performed with unpaired t-test analysis with each control treatment (a) UC82B wt and (b) non-mycorrhizal (Nm).

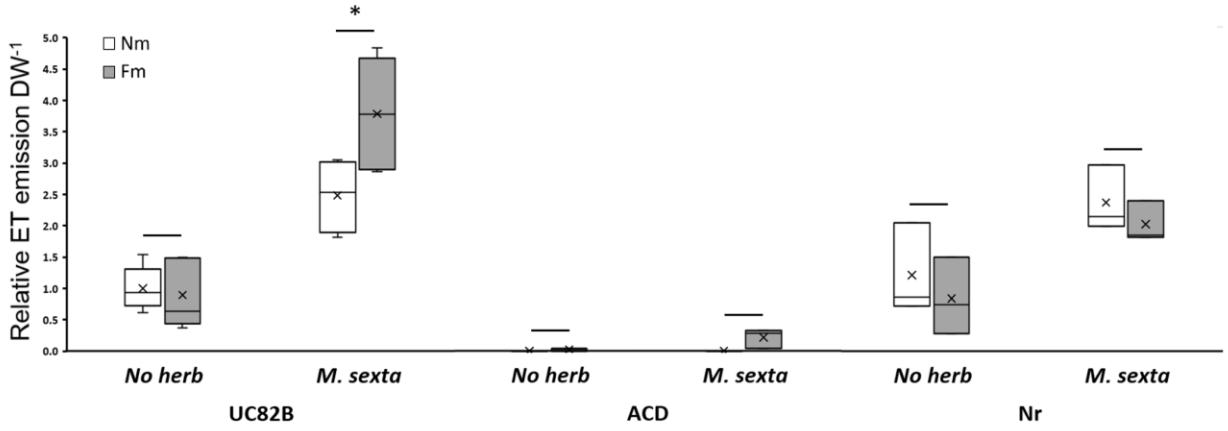

Supplemental Fig. S7. Mycorrhiza primed ET biosynthesis upon *M. sexta* herbivory is lost in ET deficient and insensitive lines. Single tomato leaflets of non-mycorrhizal (Nm) and *F. mosseae* mycorrhizal (Fm) plants in the wild-type (UC82B) or ET-deficient and insensitive lines (ACD, Nr) were incubated with *M. sexta* herbivory or without herbivory (No herb) for 3 h inside 20 mL glass vials. 1 mL of every sample was withdrawn from the vial and the area of the ethylene peak was analyzed in by gas chromatography. Boxplots of 5 biological replicates. Statistical analysis was performed with unpaired t-test analysis between each herbivory treatment. * $p<0.05$.

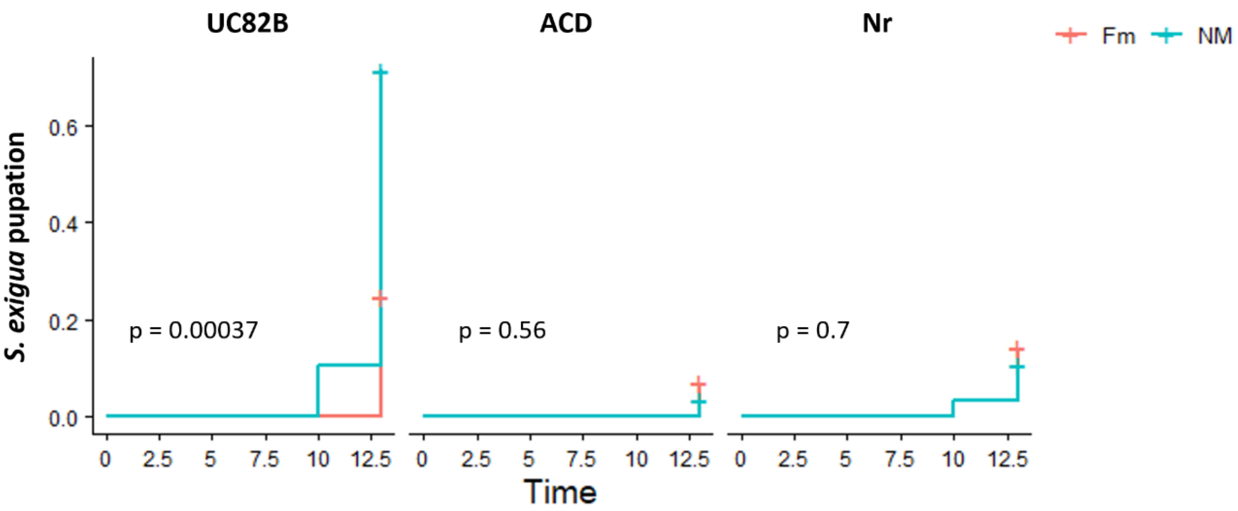

Supplemental Fig. S8. *S. exigua* pupation in non-mycorrhizal and mycorrhizal wt and ET deficient and insensitive lines. *S. exigua* larval pupation was monitored at every 2-3 days. We placed 4 3rd *S. exigua* larvae on the plant's first true leaf of non-mycorrhizal (Nm) and *F. mosseae* mycorrhizal (Fm) plants in the wild-type (UC82B) or ET-deficient and insensitive lines (ACD, Nr), and let them feed inside an entomological bag of 7 plants (n=28 larvae) per treatment. Before they had consumed the whole leaf, we moved them to the next consecutive leaf. Statistical analysis was performed with differences between curves estimated with a logrank (Mantel-Cox) test.

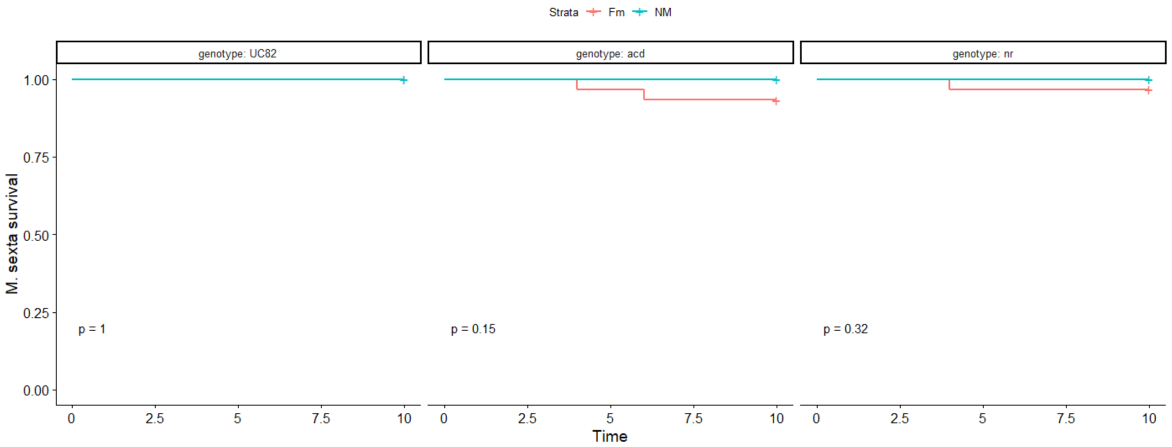

Supplemental Fig. S9. No effect on *M. sexta* mortality was observed among the genotypes. Larval performance was monitored at every 2-3 days on tomato plants of non-mycorrhizal (Nm) and *F. mosseae* mycorrhizal (Fm) plants in the wild-type (UC82B) or ET deficient and insensitive lines (ACD, Nr). We placed 3 neonate *M. sexta* larvae on the plant's first true leaf and let them feed inside an entomological bag of 10 plants (n=30 larvae) per treatment. Before they had consumed the whole leaf, we moved them to the next consecutive leaf. Differences between curves were estimated with a logrank (Mantel-Cox) test.

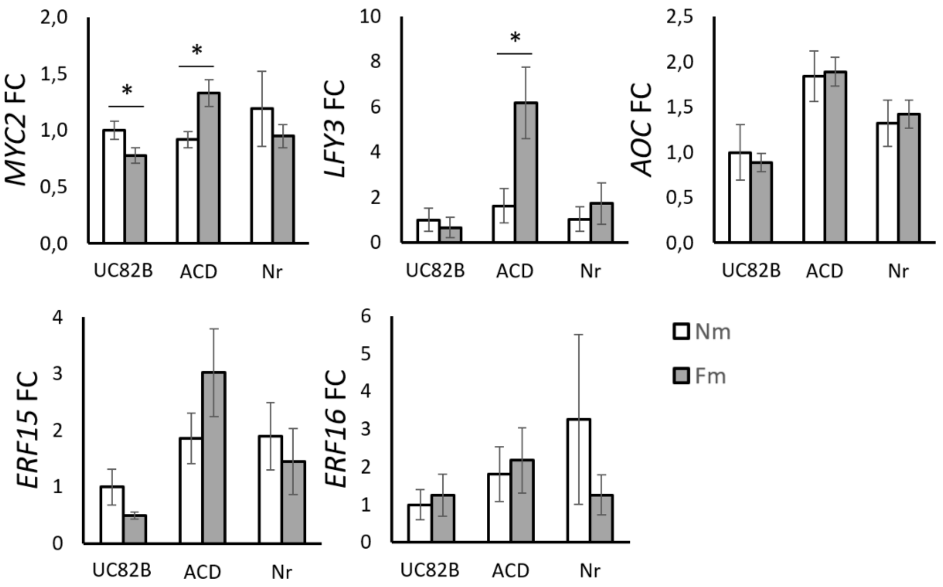

Supplemental Fig. S10. Relative expression of JA-ET related transcription factor genes in wt or ET deficient and insensitive tomato lines after 24h of *M. sexta* herbivory. *MYC2* (Solyc08g076930), *LFY3* (Solyc03g118160), *ERF15* (Solyc06g054630), *ERF16* (Solyc12g009240) and *AOC* (Solyc02g085730). Tomato plants of non-mycorrhizal (Nm) and *F. mosseae* mycorrhizal (Fm) plants in the wild-type genotype (UC82B) or ET deficient and insensitive lines (ACD, Nr) were subjected to *M. sexta* herbivory. 3 larvae were added per plant, and newly infested leaves were harvested 24 h after infestation. Data represent mean ± SEM of 6 biological replicates. Expression values were normalized using the reference gene *SlEF*. Statistical analysis was performed with unpaired t-test analysis between each herbivory treatment. * $p<0.05$.

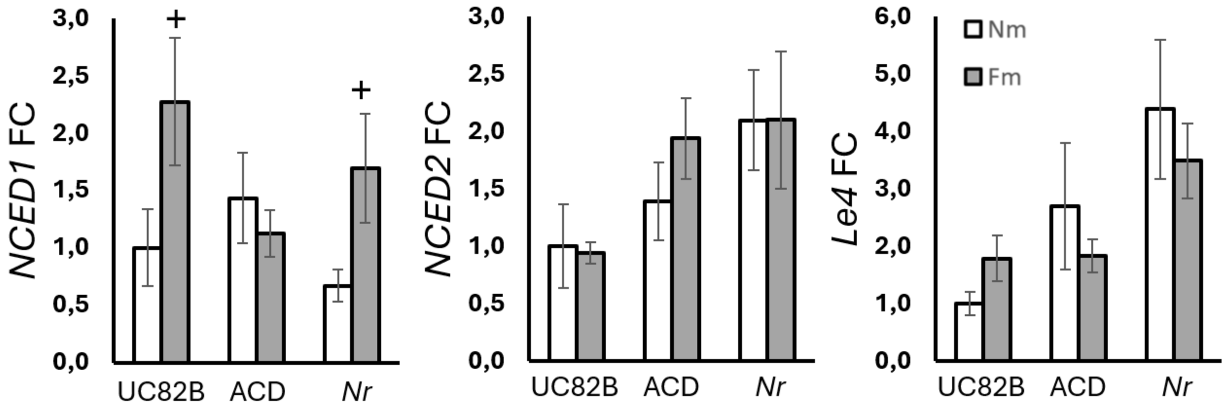

Supplemental Fig. S11. Relative expression of ABA-dependent genes in wt or ET deficient and insensitive tomato lines after 24h of *M. sexta* herbivory. *NCED1* (Solyc07g056570), *NCED2* (Solyc01g087260) and *Le4* (Solyc02g084850). Tomato plants of non-mycorrhizal (Nm) and *F. mosseae* mycorrhizal (Fm) plants in the wild-type genotype (UC82B) or ET deficient and insensitive lines (ACD, Nr) were subjected to *M. sexta* herbivory. 3 larvae were added per plant, and newly infested leaves were harvested 24 h after infestation. Data represent mean ± SEM of 6 biological replicates. Expression values were normalized using the reference gene *SlEF*. Statistical analysis was performed with unpaired t-test analysis between each herbivory treatment. + p<0.1.

Supplemental Table S1. GSEA Manually organized functional supergroups from enriched gene sets.

Functional supergroups	Enriched Gene Sets
Photosynthesis	1; 1.1; 1.1.1; 1.1.1.1; 1.1.1.2; 1.1.2; 1.1.2.2; 1.2; 1.3
Cell wall synthesis	10.1; 10.2; 10.2.1
Cell wall degradation	10.6.1
Cell wall modification	10.7
Lipid degradation	11.9; 11.9.2; 11.9.2.1
Amino acid synthesis	13.1.1; 13.2; 13.2.3
Metal handling	15; 15.2
Secondary metabolism	16
Isoprenoids	16.1.2
Phenylpropanoids	16.2
Lignin	16.2.1
Glucosinolates	16.5.1; 16.5.1.1; 16.5.1.1.1
Hormone metabolism	17
Abscisic acid metabolism	17.1
Brassinosteroid metabolism	17.3.1
Ethylene metabolism	17.5.1
Ethylene signal transduction	17.5.2
Jasmonate metabolism	17.7; 17.7.1
Tetrapyrrole synthesis	19
PR proteins	20.1.7
Stress biotic	20.1; 20.1.2; 20.1.2.1; 20.1.2.2
Stress abiotic heat	20.2.1
REDOX	21.4
Nucleotide synthesis	23.1
UDP glucosyl and glucuronosyl transferases	26.2
Cytochrome P450	26.10
Misc. Protease Inhib./Seed storage/LTP proteins	26.21
GDSL lipase	26.28
TF MYB	27.3.25
TF NAC	27.3.27
TF TCP	27.3.29
TP AP2/EREBP	27.3.3
TF WRKY	27.3.32
TF Zn finger	27.3.7
DNA chromatin structure	28.1; 28.1.3; 28.1.3.2
DNA repair	28.2
Protein amino acid activation	29.1
Ribosomal protein synthesis (prokaryotic chloroplast)	29.2.1.1; 29.2.1.1.1; 29.2.1.1.1.2
Ribosomal protein synthesis (eukaryotic)	29.2.1.2; 29.2.1.2.1; 29.2.1.2.2

Protein targeting chloroplast	29.3.3
Protein secretion	29.3.4
Protein degradation	29.5.1; 29.5.3; 29.5.9
Protein folding and assembly	29.6; 29.8
Signaling in sugar and nutrients	30.1
Receptor kinases signaling	30.2; 30.2.3
Light signaling	30.11
Calcium signaling	30.3
Cell cycle	31.3
LEA proteins	33.2
Transport. Major intrinsic proteins	34.19
Not assigned TPRs	35.1.27
PPR protein	35.1.5

Supplemental Table S2. Primers used for qPCR

Primer	Sequence	Solyc	Reference
SlEF-1-F	GATTGGTGGTATTGGAACTGTC	Solyc06g009960	Rotenberg et al., 2006
SlEF-1-R	AGCTTCGTGGTGCATCTC		
SlActin-F	TTGCTGACCGTATGAGCAAG	Solyc11g005330	Yan et al., 2013
SlActin-R	GGACAATGGATGGACCAGAC		
SlCAC-F	CCTCCGTTGTGATGTAACTGG	Solyc08g006960	Expósito-Rodríguez et al., 2008
SlCAC-R	ATTGGTGGAAAGTAACATCATCG		
LOXD-F	GACTGGTCCAAGTTCACGATCC	Solyc03g122340	Uppalapati et al., 2005
LOXD-R	ATGTGCTGCCAATATAAATGGTTCC		
AOS1-F	CACCTGTTAAACAAGCGAAAC	Solyc04g079730	López-Ráez et al., 2010
AOS1-R	GACCTGGTGGCATGTTCGT		
AOC-F	GCACGAAGAAGAGAAGAAAGGAGAT	Solyc02g085730	Uppalapati et al., 2005
AOC-R	CGGTGACGGCTAGGTAAGTTTC		
OPR3-F	TTGGCTTAGCAGTTGTTGAAAG	Solyc07g007870	Uppalapati et al., 2005
OPR3-R	TACGTATCGTGGCTGTGTTACA		
PPOF-F	CGGAGTTTGCAGGGAGTTATAC	Solyc08g074620	Alba et al., 2015
PPOF-R	TTGATCTCCACACTTTCAATGG		
TD-F	AGCTCAAACACACGCGCTGGA	Solyc09g008670	Yan et al., 2013
TD-R	AACCCCCACCACCAACAGGT		
MC-F	GAGAATTTCAAGGAAGTTCAA	Solyc00g071180	Uppalapati et al., 2005
MC-R	GGCTTTATTTCACACAGAGATA		
LapA-F	ATCTCAGGTTTCCTGGTGGAAGGA	Solyc12g010020	Yan et al., 2013
LapA-R	AGTTGCTATGGCAGAGGCAGAG		
ACS6-F	GGGTTTCCTGGATTTAGGGT	Solyc08g008100	Ibort, 2017
ACS6-R	GGTACTCAGTGAAATAGTCGA		
ERF-F	GAGATCCTCTGGAGTCGAAAT	Solyc02g070040	Wang et al., 2020
ERF-R	ACTTGACTCTTCTTGCTGTAAT		
ACO1-F	AAGGGACTCCGCGCTCATA	Solyc07g049530	Chersicola et al., 2017
ACO1-R	CAAGTTGGTCACCAAGGTTAACC		
ACO4-like-F	CCCAGTTTCTTCATCCACTCA	Solyc04g007980	Satková et al., 2017
ACO4-like-R	AGAAAAGTCGACGACGGGTAT		
WRKY39-F	GCTCCTACCTGTCCCGTTAA	Solyc03g116890	This work
WRKY39-R	CGGGTTAAATCGGCTAGACG		
WRKY40-F	GCCTCGTCAAAAAGTCCTGAAAC	Solyc06g068460	This work
WRKY40-R	CCCCTGCCTCATTTTTCACCA		
CBF1-F	GTGACTTCGTGGATGAGGAG	Solyc03g026280	Fang et al., 2021
CBF1-R	AGGCATCAGTTTCCACACAA		
CBF2-F	TTCGATCGGAAGAAGTTTCA	Solyc03g124110	Fang et al., 2021
CBF2-R	CAAGTAATCCTGGCATGGAA		
ERF15-F	ACAGGCTGTAGCAGCTAGAT	Solyc06g054630	Hu et al., 2021
ERF15-R	TATTTCCAATATTGCCCTCG		
ERF16-F	GCTGCTAAAGCATTTGACGC	Solyc12g009240	Hu et al., 2021
ERF16-R	GTCATCGTCCTTCCGTTCCT		

MYC2-F	ATCTCGAGGCTTCAGTGGTG	Solyc08g076930	This work
MYC2-R	ACGTGATTCAATGGCTCCTC		
LFY3-F	GCTCCCAACATCATCCTACTCC	Solyc03g118160	Cui et al., 2020
LFY3-R	CGCTTTGATACCGTACCTCTCTC		
NCED1-F	ACCCACGAGTCCAGATTTC	Solyc07g056570	López-Ráez et al., 2010
NCED1-R	GGTTCAAAAAGAGGGTTAGC		
NCED2-F	GCCAAAAGTATCTGGATTTGC	Solyc01g087260	This work
NCED2-R	TTTCCATGTCTTCTCGTCGTG		
Le4-F	ACTCAAGGCATGGGTACTGG	Solyc02g084850	Herrera-Medina et al., 2007
Le4-R	CCTTCTTTCTCCTCCCACCT		

Supplemental Table S3. RNA-seq DEGs overview. Tomato leaves of non-mycorrhizal plants (Nm) and mycorrhizal plants inoculated with *F. mosseae* (Fm) were subjected to 24h of herbivory by the generalist *S. exigua* (NmSe, FmSe) and the specialist *M. sexta* (NmMs, FmMs). Data shown represent DEGs with an FDR<0.05. Differential expression analysis was performed in R using the DESeq2 package.

	**Down**	**Up**
Fm vs Nm	25	32
NmSe vs Nm	2832	3340
NmMs vs Nm	1978	2556
FmSe vs Nm	2733	2921
FmMs vs Nm	3292	3598
NmSe vs NmMs	0	0
FmSe vs Fm	1801	2065
FmMs vs Fm	2243	2647
FmSe vs NmSe	0	0
FmMs vs NmMs	12	22
FmSe vs FmMs	2	0
